# Factors associated with development and distribution of granular/fuzzy astrocytes in neurodegenerative diseases

**DOI:** 10.1111/bpa.12843

**Published:** 2020-05-06

**Authors:** Tomoko Miki, Osamu Yokota, Takashi Haraguchi, Hideki Ishizu, Masato Hasegawa, Takeshi Ishihara, Shu‐ichi Ueno, Shintaro Takenoshita, Seishi Terada, Norihito Yamada

**Affiliations:** ^1^ Department of Neuropsychiatry Okayama University Graduate School of Medicine Dentistry and Pharmaceutical Sciences Okayama Japan; ^2^ Department of Psychiatry Kinoko Espoir Hospital Okayama Japan; ^3^ Department of Laboratory Medicine and Pathology Zikei Institute of Psychiatry Okayama Japan; ^4^ Department of Neurology National Hospital Organization Minami‐Okayama Medical Center Okayama Japan; ^5^ Dementia Research Project Tokyo Metropolitan Institute of Medical Science Tokyo Japan; ^6^ Department of Psychiatry Kawasaki Medical School Okayama Japan; ^7^ Department of Neuropsychiatry Ehime University Graduate School of Medicine Ehime Japan

**Keywords:** aging‐related tau astrogliopathy, argyrophilic grain, granular/fuzzy astrocyte, primary age‐related tauopathy, tau, tufted astrocyte

## Abstract

Granular/fuzzy astrocytes (GFAs), a subtype of “aging‐related tau astrogliopathy,” are noted in cases bearing various neurodegenerative diseases. However, the pathogenic significance of GFAs remains unclear. We immunohistochemically examined the frontal cortex, caudate nucleus, putamen and amygdala in 105 cases composed of argyrophilic grain disease cases (AGD, N = 26), and progressive supranuclear palsy (PSP, N = 10), Alzheimer’s disease (AD, N = 20) and primary age‐related tauopathy cases (PART, N = 18) lacking AGD, as well as 31 cases bearing other various neurodegenerative diseases to clarify (i) the distribution patterns of GFAs in AGD, and PSP, AD and PART lacking AGD, (ii) the impacts of major pathological factors and age on GFA formation and (iii) immunohistochemical features useful to understand the formation process of GFAs. In AGD cases, GFAs consistently occurred in the amygdala (100%), followed by the putamen (69.2%) and caudate nucleus and frontal cortex (57.7%, respectively). In PSP cases without AGD, GFAs were almost consistently noted in all regions examined (90–100%). In AD cases without AGD, GFAs were less frequent, developing preferably in the putamen (35.0%) and caudate nucleus (30.0%). PART cases without AGD had GFAs most frequently in the amygdala (35.3%), being more similar to AGD than to AD cases. Ordered logistic regression analyses using all cases demonstrated that the strongest independent factor of GFA formation in the frontal cortex and striatum was the diagnosis of PSP, while that in the amygdala was AGD. The age was not significantly associated with GFA formation in any region. In GFAs in AGD cases, phosphorylation and conformational change of tau, Gallyas‐positive glial threads indistinguishable from those in tufted astrocytes, and the activation of autophagy occurred sequentially. Given these findings, AGD, PSP, AD and PART cases may show distinct distributions of GFAs, which may provide clues to predict the underlying processes of primary tauopathies.

## Introduction

Currently, the pathological diagnosis of various tauopathies is based on the presence of disease‐specific neuronal and glial inclusions in which phosphorylated tau protein is abnormally aggregated, as well as their anatomical distribution. Neurofibrillary tangles (NFTs) are the most common non‐disease‐specific tau‐positive lesions, and historically, several studies have attempted to define several diseases by the anatomical distributions of NFTs alone visualized by early silver impregnation methods. Thereafter, argyrophilic astrocytic lesions demonstrated by a sensitive silver stain, the Gallyas method, that is, tufted astrocytes (TAs) (35, 57, 58, 77) and astrocytic plaques (16, 35), were found to have diagnostic value in progressive supranuclear palsy (PSP) and corticobasal degeneration (CBD). The Gallyas method also demonstrated the thorn‐shaped astrocytes that are found in cases having various pathological conditions (26). Subsequently, the accumulation of phosphorylated tau in these astrocytic lesions was reported (16, 26, 57).

Besides thorn‐shaped astrocytes, another morphological subtype of tau‐positive non‐disease‐specific astrocytic lesions has been reported. The astrocytic lesions have a small amount of tau accumulation in the perinuclear regions of astrocytes, as well as fine or fuzzy granular tau accumulation in astrocytic processes. Botez *et al* first described this subtype of astrocytic lesions in the amygdala in brains with argyrophilic grain disease (AGD) and called them “bush‐like astrocytes” (4). They also noticed that these astrocytic lesions were not stained by the Gallyas method. Thereafter, morphologically and immunohistochemically similar tau‐positive astrocytic lesions were also reported using different terms, for example, diffuse granular tau immunoreactivity in astrocytic processes (42), diffuse granular tau immunopositivity along astrocytic processes (41), fine granular tau immunoreactivity in astrocytic processes (17), astrocytes with finely tau immunoreactive processes (76), TA‐like astrocytic lesions (27) and granular‐shaped astrocytes (80). In a recently proposed classification of tau‐positive astrocytic lesions, these tau‐positive astrocytic lesions were called “granular/fuzzy astrocytes (GFAs)” as a subtype of “aging‐related tau astrogliopathy (ARTAG)” (38).

GFAs preferably occur in the gray matter, that is, the cerebral cortex and nuclei rather than the white matter (37, 38). GFAs are basically Gallyas‐negative, although accumulated tau only in the cytoplasm of astrocytes can rarely show weak argyrophilia (38). GFAs can be found in elderly cases having heterogeneous clinical and pathological bases (17, 27, 41, 42), including PSP (27, 44, 80), Alzheimer’s disease (AD) (44), primary age‐related tauopathy (PART) (44), CBD (44), Pick’s disease (44) and chronic traumatic encephalopathy (18). It is not always easy to evaluate the effect of each pathology on GFA formation because several tauopathies often coexist in a single case. Especially, given that AGD increases in frequency with age (9) and might be associated with the development of GFAs (4), the potential effect of AGD on the frequency of GFAs in other tauopathies needs to be carefully evaluated. However, although only a few previous studies have examined the relationship between various tauopathies and ARTAG (ie, thorn‐shaped astrocytes and GFAs) (43, 44), as far as we know, the frequency and distribution of GFAs by specific anatomical regions in pathological conditions common in the elderly, that is, AGD, PSP without AGD, AD without AGD or PART without AGD, have not been examined.

The aims of the study described here were to examine (i) the frequency and distribution of GFAs in AGD cases, as well as PSP, AD and PART cases lacking AGD, respectively, (ii) the impacts of major pathological factors and the age on GFA formation in regions in which GFAs preferably occur and (iii) immunohistochemical findings useful to understand the formation process of GFAs. To address these issues, we first semiquantitatively examined GFAs, as well as TAs, which are potentially related to GFA formation (27), in the frontal cortex, caudate nucleus, putamen and amygdala in AGD, PSP, AD and PART cases, as well as cases of various other degenerative diseases. Then, after performing univariate and multivariate analyses to assess the effects of factors on the development of GFAs by region in all cases, we examined the immunohistochemical features of GFAs in AGD cases using phosphorylation‐ or conformational change‐specific anti‐tau, anti‐ubiquitin and anti‐p62 antibodies as well as the Gallyas method. In this paper, we demonstrate that GFAs may occur with disease‐specific distribution patterns in cases of AGD, PSP without AGD, AD without AGD and PART without AGD, respectively, and that age may not be always associated with GFA formation. In addition, we demonstrate that the increase of immunoreactivities of phosphorylation‐ and conformation‐dependent anti‐tau antibodies in GFAs, the occurrence of Gallyas‐positive glial threads in GFAs and the activation of autophagy may occur sequentially in AGD cases.

## Materials and Methods

### Subjects

A total of 1125 autopsy cases were registered in the database at the Department of Neuropsychiatry, Okayama University Graduate School of Medicine, Dentistry and Pharmaceutical Sciences as of the end of December 2017. All cases died in psychiatric hospitals or neurological departments of general hospitals. We first selected 202 cases that were recently assessed using modern standardized methods including a panel of immunohistochemistry, modified Bielschowsky silver stain and Gallyas‐Braak silver stain from 2001 to 2017 for the selection of AGD, PSP, AD and PART cases. AGD cases (N = 26) that lacked any other primary tauopathies except for PART [ie, AD, PSP, CBD, globular glial tauopathy (GGT), diffuse NFTs with calcification (DNTC), Pick’s disease and frontotemporal lobar degeneration with tau gene mutation (FTLD‐MAPT)] were selected. Likewise, PSP (N = 10) cases that lacked any other primary tauopathies except for PART were selected. AD (N = 20) cases without any other tauopathies were then selected. Definite PART cases without any other degenerative disease [N = 5; Braak & Braak stage (Braak stage) I–IV, Thal phase 0] were also selected from the 202 cases.

Then, to evaluate the effect of age and common pathological factors (ie, Braak stage, Thal phase, the presence of Lewy bodies and TDP‐43 pathology) on the development of GFAs, 19 cases of other various tauopathies, that is, CBD (N = 7), GGT (N = 2), DNTC (N = 5), Pick’s disease (N = 3), FTLD‐MAPT (N = 1) and postencephalitic parkinsonism (N = 1), as well as 25 non‐tauopathy cases lacking any other tauopathies except for PART, that is, myotonic dystrophy (N = 4), multiple system atrophy (MSA, N = 6), frontotemporal lobar degeneration with TDP‐43‐positive inclusions (FTLD‐TDP, N = 3), amyotrophic lateral sclerosis with TDP‐43‐positive inclusions (ALS‐TDP, N = 4), FTLD with fused in sarcoma (FUS)‐positive inclusions (FTLD‐FUS, N = 3), Huntington’s disease (N = 1) and dentatorubral‐pallidoluysian atrophy (DRPLA, N = 4), were selected from our 1125 cases.

Among the 25 non‐tauopathy cases, 13 cases also had PART: nine had definite PART (Braak stage I–IV, Thal phase 0), and four had possible PART (Braak stage I–IV, and Thal phases 1–2). Therefore, we made a PART group (N = 18) by combining these additional 13 PART cases and the first five definite PART cases without any other degenerative diseases (Supporting File S1).

The demographic data of a total of the 105 cases that we eventually examined in the present study are shown in Table 1. In this study, except for AGD and CBD cases, we selected only cases that lacked argyrophilic grains in any anatomical region. Because all of our CBD cases had various degrees of AGD, they were exceptionally included in this study despite the coexistence of AGD. Regarding PSP pathology, several previous studies including ours suggested that AGD cases often had small numbers of NFTs and TAs in the basal ganglia and brain stem nuclei, distribution of which is reminiscent of that in PSP cases (27, 64). While TAs are considered to be specific to PSP, whether all TAs observed in cases of other diseases mean an early change of PSP remains unclear. Therefore, in the present study, even when cases had a few Gallyas‐ or AT8‐positive TAs, they were not diagnosed as PSP when lacking NFTs that met the pathological criteria of PSP (25). For example, when an AGD case had a few AT8‐positive TAs but lacked sufficient NFTs that fit the pathological criteria of PSP, the case was classified into an AGD group.

**Table 1:** Demographic data of all 105 subjects

Pathological diagnosis	All	AGD	PSP	AD	PART^†^	PART without other degenerative diseases	CBD	GGT	DNTC	Pick’s disease	FTLD‐MAPT (P301L)	Postencephalitic parkinsonism	Myotonic dystrophy	MSA	FTLD‐TDP	ALS‐TDP	FTLD‐FUS	Huntington’s disease	DRPLA
Number of cases (N)	105	26	10	20	18	(5)	7	2	5	3	1	1	4	6	3	4	3	1	4
Male/female (N)	60/45	13/13	8/2	9/11	14/4	(5/0)	5/2	0/2	2/3	1/2	1/0	1/0	2/2	3/3	2/1	4/0	2/1	0/1	2/2
Age at death (y) (mean ± SD)	70.4 ± 11.8	77.0 ± 11.9	70.0 ± 7.9	80.9 ± 8.7	66.3 ± 9.7	(69.8 ± 4.4)	62.3 ± 12.1	68.0 ± 12.7	60.6 ± 13.5	74.0 ± 9.0	60	72	58.0 ± 7.1	63.7 ± 7.5	72.7 ± 9.5	65.3 ± 10.2	64.5 ± 3.5	59	51.0 ± 10.2
(range)	42‐95	61‐94	61‐84	63‐95	52‐82	(63‐74)	45‐85	59‐77	48‐75	65‐83	‐	‐	50‐67	58‐76	63‐82	66‐77	45‐62	‐	42‐65
Brain weight (g, mean ± SD)	1154 ± 224.4	1180.5 ± 173.9	1236.7 ± 246.8	1072.0 ± 188.8	1159.0 ± 301.3	(1367.4 ± 152.6)	1238.6 ± 150.2	1120.0 ± 198.0	946.7 ± 126.6	863.3 ± 225.9	970	1210	1117.0 ± 115.9	1178.0 ± 192.0	933.0 ± 198.0	1438.0 ± 92.3	1100.0 ± 203.0	610	1017.5 ± 263.2
Braak stage (6), median (25t^h^‐75^th^ percentiles)	2 (1‐4)	2 (1‐2)	1 (1‐2)	5 (5‐6)	1 (1‐1.5)	2 (1‐2)	2 (1‐2.5)	1.5 (0.25‐0.75)	6 (6‐6)	2 (1‐2)	1 (1‐1)	5 (0‐0)	3 (2‐4)	2 (1.25‐3.5)	1 (0.5‐1)	2.5 (1‐4)	0 (0‐0)	1 (1‐1)	0 (0‐0.5)
Thal phase (69), median (25^th^‐75^th^ percentiles)	0 (0‐3)	1 (0‐3)	1 (0‐1.75)	5 (4‐5)	0 (0‐0.5)	0 (0.0)	0 (0‐1)	0.5 (0.25‐0.75)	0 (0‐0)	1 (0.5‐2.5)	0 (0‐0)	4 (0‐0)	0 (0‐0)	0.5 (0‐1.75)	1 (0.5‐1)	0 (0‐0.25)	0 (0‐0)	0 (0‐0)	0 (0‐0)
AGD stage (63), median (25^th^‐75^th^ percentiles)	0 (0‐1)	2 (1‐2)	0 (0‐0)	0 (0‐0)	0 (0‐0)	0 (0‐0)	1 (1‐2.5)	0 (0‐0)	0 (0‐0)	0 (0‐0)	0 (0‐0)	0 (0‐0)	0 (0‐0)	0 (0‐0)	0 (0‐0)	0 (0‐0)	0 (0‐0)	0 (0‐0)	0 (0‐0)
Cases having LBD [N, (%)]	25 (23.8)	4 (15.4)	2 (20.0)	11 (55.0)	1 (5.6)	0 (0.0)	1 (14.3)	1 (50.0)	4 (80.0)	1 (33.3)	0 (0.0)	0 (0.0)	0 (0.0)	0 (0.0)	1 (33.3)	0 (0.0)	0 (0.0)	0 (0.0)	0 (0.0)
Cases having TDP‐43 pathology [N, (%)]	33 (31.4)	6 (23.1)	4 (40.0)	13 (65.0)	6 (33.3)	0 (0.0)	1 (14.3)	0 (0.0)	1 (20.0)	1 (33.3)	0 (0.0)	0 (0.0)	0 (0.0)	0 (0.0)	3 (100.0)	4 (100.0)	0 (0.0)	0 (0.0)	0 (0.0)

Abbreviations: AD = Alzheimer’s disease; AGD = argyrophilic grain disease; CBD = corticobasal degeneration; DNTC = diffuse neurofibrillary tangles with calcification; DRPLA = dentatorubral‐pallidoluysian atrophy; FTLD‐FUS = frontotemporal lobar degeneration with FUS‐positive inclusions; FTLD‐MAPT = frontotemporal lobar degeneration with tau gene mutation; FTLD‐TDP = frontotemporal lobar degeneration with TDP‐43‐positive inclusions; GGT = globular glial tauopathy; LBD = Lewy body disease; MSA = multiple system atrophy; N = number; NFT = neurofibrillary tangles; PART = primary age‐related tauopathy; PSP = progressive supranuclear palsy; SD = standard deviation.

^†^ The PART group is composed of five PART cases without any other degenerative diseases and 13 PART cases with some non‐tauopathy (e.g., FTLD‐TDP, ALS‐TDP, MSA, DRPLA and Huntington’s disease). Detailed demographic data of the PART group are shown in Supporting File S1.

Autopsies were carried out after informed consent was obtained from family members, and all experiments in this study were approved by the ethical committees of the Okayama University Graduate School of Medicine, Dentistry and Pharmaceutical Sciences, the National Hospital Organization Minami‐Okayama Medical Center, Zikei Institute of Psychiatry and the Tokyo Metropolitan Institute of Medical Science.

The criteria of each pathological diagnostic category were as follows: AGD was diagnosed based on Gallyas‐positive grains in the limbic system with and without the temporal cortex (7) and the absence of any other primary tauopathies except for PART (13) [ie, NFTs with Braak stage I–IV (6, 8) and no or minimal Aβ deposits with Thal phase 0–2 (69)]. The diagnosis of PSP was based on the presence of both Gallyas‐positive TAs and AT8‐positive NFTs whose quantity and distribution met the pathological criteria of either typical or atypical PSP (25). The AD group was composed of cases having intermediate or high level AD pathology (Braak stage V–VI, Thal phase 3–5) (52). Cases of CBD met the pathological criteria and had Gallyas‐positive AT8‐positive astrocytic plaques in the striatum and frontal cortex (15). Cases of GGT (N = 2) had numerous 4‐repeat (4R) tau‐positive 3‐repeat (3R) tau‐negative globular glial inclusions that correspond to type I (N = 1) and type II (N = 1) histologies (1). DNTC was diagnosed based on severe NFTs (Braak stage VI), the absence of or minimal Aβ deposits (ie, Thal phase 0–1), Fahr‐type calcification and circumscribed temporal lobe atrophy (31, 36, 66, 78). Pick’s disease was defined by the presence of 3R tau‐positive 4R tau‐negative Pick bodies (53, 79). Previously reported cases of frontotemporal lobar degeneration due to P301L tau mutation (50) and postencephalitic parkinsonism (22) were also included. Because, the case of postencephalitic parkinsonism showed distinct clinical and pathological findings (30), it was not included in the AD group although it had severe NFTs and Aβ deposits (Braak stage V, Thal phase 4). MSA was diagnosed based on Gallyas‐positive α‐synuclein‐positive Papp–Lantos inclusions and tissue degeneration with a distinctive distribution (61, 74). Diagnoses of FTLD‐TDP (2, 12, 47, 56) and ALS‐TDP were based on TDP‐43 pathology and neuronal loss with a distinct distribution and the absence of other primary neurodegenerative diseases. FTLD‐FUS was composed of two cases of basophilic inclusion body disease and one ALS case with the *FUS* mutation (12, 54, 55). The diagnoses of DRPLA and Huntington’s disease were pathologically and genetically confirmed. Myotonic dystrophy cases had a diagnosis of DM1 by genetic and/or clinical criteria (21, 71).

### Conventional neuropathological examination and immunohistochemistry

A standardized neuropathological evaluation was done on all subjects. Brain tissue samples were fixed post‐mortem with 10% of formaldehyde and embedded in paraffin. Ten‐µm‐thick sections from the frontal, temporal, parietal, occipital, insular and cingulate cortices, hippocampus, amygdala, basal ganglia, midbrain, pons, medulla oblongata and cerebellum in the left hemisphere were prepared. For the standardized neuropathological assessment, sections were stained with hematoxylin‐eosin and Klüver‐Barrera stains, and selected regions stained with modified Bielschowsky silver and Gallyas methods.

Then, formalin‐fixed paraffin sections were cut at six μm thickness from selected regions from all cases for the standard immunohistochemical evaluation of representative neurodegenerative changes. Deparaffinized sections were incubated with 1% of H_2_O_2_ in methanol for 30 minutes to eliminate endogenous peroxidase activity, and washed in phosphate‐buffered saline (PBS, pH 7.4). After blocking with 10% of normal serum, sections were incubated overnight at 4°C with primary antibodies (Table 2). When using anti‐tau, anti‐ubiquitin, anti‐p62, anti‐α‐synuclein and anti‐TDP‐43 antibodies, sections were autoclaved for 10 minutes in 10 mM of sodium citrate buffer at 121°C for antigen retrieval. When using 12B2, RD4, RD3 and T22, sections were autoclaved for 10 minutes in 10 mM of sodium citrate buffer at 121°C and treated with 70% of formic acid for 10 minutes. After three 5‐min washes in PBS, sections were incubated in biotinylated secondary antibody for 30 minutes, and then, in avidin‐biotinylated horseradish peroxidase complex (ABC Elite kit, Vector, Burlingame, CA, USA) for 60 minutes. After three 5‐min washes in PBS, the peroxidase labeling was visualized with 3,3’‐diaminobenzidine (DAB) as the chromogen. Sections were lightly counterstained with hematoxylin.

**Table 2:** Antibodies used in this study

Antibody	Species/type	Dilution	Epitope	Source
AT8	Mouse/monoclonal	1:1000	Tau phosphorylated at Ser 202	Innogenetics
AT100	Mouse/monoclonal	1:500	Tau phosphorylated at Ser 212 and Thr 214	Innogenetics
AT180	Mouse/monoclonal	1:500	Tau phosphorylated at Thr 231	Innogenetics
AT270	Mouse/monoclonal	1:500	Tau phosphorylated at Thr 181	Innogenetics
PHF‐1	Mouse/monoclonal	1:250	Tau phosphorylated at Ser 396 and Ser 404	(20)
Alz‐50	Mouse/monoclonal	1:250	Tau epitope at aa 5‐15	(5)
MC‐1	Mouse/monoclonal	1:250	Conformation‐dependent tau epitope within aa 312‐322	(32)
T46	Mouse/monoclonal	1:500	Phosphorylation independent epitope in aa 404‐441 of human tau	Invitrogen
T22	Rabbit/polyclonal	1:500	Tau oligomer	(46)
RD3	Mouse/monoclonal	1:2000	3 repeat tau‐specific anti‐tau antibody	Merck Millipore (67)
RD4	Mouse/monoclonal	1:100	4 repeat tau‐specific anti‐tau antibody	Merck Millipore (67)
Anti‐4R tau	Rabbit/polyclonal	1:2000	4 repeat tau‐specific anti‐tau antibody	Cosmo Bio
MAB1510	Mouse/monoclonal	1:500	Ubiquitin	Merck Millipore
p62‐N	Guinea pig/polyclonal	1:200	N‐terminus of p62 protein	Progen Biotechnik
p62‐C	Guinea pig/polyclonal	1:500	C‐terminus of p62 protein	Progen Biotechnik
12B2	Mouse/monoclonal	1:100	Aβ(11–28)	IBL
psyn#64	Mouse/monoclonal	1:5000	Phosphorylated α‐synuclein	Wako
pS409/410‐2	Rabbit/polyclonal	1:5000	Phosphorylated TDP‐43	Cosmo Bio
HPA008784	Rabbit/polyclonal	1:200	FUS	Sigma‐Aldrich
SMI31	Mouse/monoclonal	1:5000	Phosphorylated neurofilament	Sternberger
GFAP	Rabbit/polyclonal	1:100	Glial fibrillary acidic protein	Dako
CD68	Mouse/monoclonal	1:500	CD68	Dako
Iba1	Rabbit/polyclonal	1:1000	C‐terminus of Iba1	Wako
3F4	Mouse/monoclonal	1:1000	Prion protein	Covance

AGD was diagnosed based on the presence of Gallyas‐, AT8‐ and RD4‐positive argyrophilic grains, and the distribution was assessed using sections stained with the Gallyas method and the staging system reported by Saito *et al* (63). Other major degenerative changes, that is, NFTs (AT8, Gallyas method) (6), Aβ deposits (12B2) (69), neuritic plaques (modified Bielschowsky silver method) (51), Lewy bodies (psyn#64) (49, 75), TAs (Gallyas method, AT8, RD4) (1), astrocytic plaques (Gallyas method, AT8, RD4) (1), Pick bodies (AT8, RD3) (28), TDP‐43 accumulation (pS409/410‐2) (10, 11, 12, 33, 34, 47), globular glial inclusions (Gallyas method, AT8, RD4) (1, 40, 62) and FUS‐positive pathology (HPA008784) (12, 54, 55) were also evaluated in all subjects using established criteria, respectively.

### Semiquantitative assessment of GFAs and TAs

It was originally reported that GFAs were frequently found in the amygdala in AGD cases (4). Our previous study revealed that GFAs may be also frequent in the frontal cortex and striatum in AGD and PSP cases (27). In addition, it was reported that these regions may be potentially significant sites to understand the progression pattern of GFAs (44). Therefore, in the present study, we semiquantitatively evaluated the density of GFAs in the superior frontal gyrus, caudate nucleus, putamen and amygdala in all subjects using the staging system: stage 0, no lesion; stage 1, one or more lesions in the anatomical region but less than one lesion per × 200 visual field; stage 2, one lesion per × 200 visual field; stage 3, 2 to 10 lesions per × 200 visual field; stage 4, over 11 lesions per × 200 visual field. The grades were determined separately by two investigators (TM and OY) who did not know whether the case had any pathological data. If there was a discrepancy between the results, the two researchers reviewed the tissues and decided the final grade by discussion.

In our previous study, some AGD cases had a few astrocytic lesions that bore both tau‐positive granular components like GFAs and Gallyas‐positive glial threads like TAs (27). In PSP cases, TAs were reported to preferentially develop in the frontal cortex and striatum (3, 24, 29). Therefore, in this study, to examine the relationship between GFAs and TAs, TAs were additionally evaluated in the superior frontal gyrus, caudate nucleus, putamen and amygdala in all cases on AT8‐ and Gallyas‐stained sections, respectively, using the staging system for GFAs.

**Figure 1:**
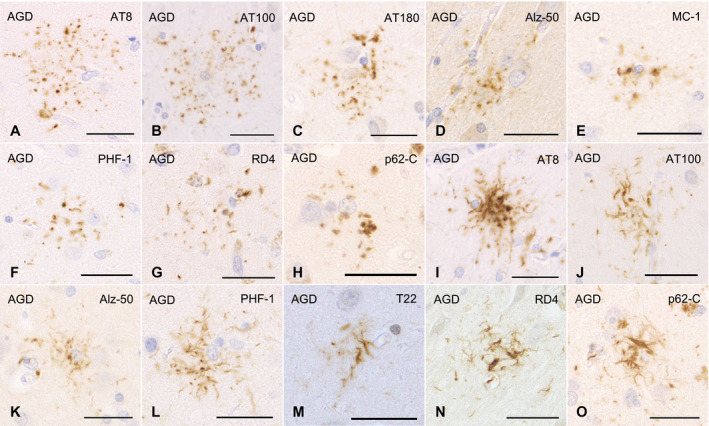
GFAs (**A**–**H**) and TAs (**I**–**O**) in AGD cases. GFAs are labeled with various anti‐tau antibodies, that is, AT8 (**A**), AT100 (**B**), AT180 (**C**), Alz‐50 (**D**), MC‐1 (**E**), PHF‐1 (**F**) and RD4 (**G**). TAs are also immunostained with AT8 (**I**), AT100 (**J**), Alz‐50 (**K**), PHF‐1 (**L**), T22 (**M**) and RD4 (**N**). GFAs and TAs are also visualized with p62‐C (**H**, **O**). (**A**, **E**, **F**) The caudate nucleus, (**B**–**D**, **G**, **I**–**O**) frontal cortex, (**H**) putamen. All scale bars = 30 μm.

To secure the reproducibility of results, we regarded tau‐positive astrocytic lesions as GFAs only when the lesions had the typical morphological features of GFAs (Figure 1A–G) originally reported in AGD cases (4) and shown in the consensus paper on ARTAG (38). In addition, because it is difficult to sharply distinguish “GFAs with Gallyas‐positive glial threads” from “Gallyas‐positive TAs” on Gallyas‐stained sections, we counted astrocytic lesions having Gallyas‐stained glial threads as TAs irrespective of the quantity of threads.

### Double immunohistochemistry and double staining of AT8 immunohistochemistry and Gallyas method

To examine whether GFAs occur in association with Aβ deposits, double immunostaining with AT8 and 12B2 was done in all cases that had Aβ deposits in the frontal cortex, striatum and/or amygdala. Primary antibody labeling in the first cycle (AT8) was detected in the same way as single staining using the DAB reaction. Then, primary antibody labeling in the second cycle (12B2) was also detected in the same way as single staining, and the reaction was visualized with Vector Blue Alkaline Phosphatase Substrate (Vector Laboratories, Inc.) as the chromogen.

Double staining using the Gallyas method and AT8 immunohistochemistry was also done in representative AGD and PSP cases to examine whether GFAs actually have TA‐like Gallyas‐positive glial threads. Sections were first stained by the Gallyas method, followed by immunostaining with AT8. The peroxidase labeling was visualized with Vector Red Alkaline Phosphatase Substrate (Vector Laboratories, Inc.) as the chromogen. Sections were lightly counterstained with hematoxylin.

### Confocal laser scanning microscopy

Double‐labeling immunofluorescence was performed with the combination of phosphorylation‐dependent anti‐tau antibody (AT8, mouse, monoclonal, 1:500) and anti‐4R tau antibody (anti‐4R tau, rabbit, polyclonal, 1:300, Cosmo Bio Co., Tokyo, Japan), and the combination of phosphorylation‐dependent anti‐tau antibody (AT8, mouse, monoclonal, 1:500) and anti‐p62‐C antibody (p62‐C, guinea pig, polyclonal, 1:200, Progen, Heidelberg, Germany). Sections from the frontal cortex and caudate nucleus in representative AGD and PSP cases having TAs and GFAs were autoclaved for 10 minutes in 10 mM of sodium citrate buffer at 120°C. Following washing in PBS, nonspecific antibody binding was blocked with a 5% of skim‐milk buffer, and sections were incubated overnight with a mixture of the two primary antibodies at 4°C. After washing in PBS, sections were incubated for 1 h at room temperature with a mixture of two fluorescence‐labeled secondary antibodies as follows: Alexa Fluor 488 goat anti‐mouse IgG (1:200, Thermo Fisher Scientific, Waltham, MA), Alexa Fluor 594 goat anti‐rabbit IgG (1:200, Thermo Fisher Scientific) or Alexa Fluor 647 goat anti‐guinea pig IgG (1:200, Thermo Fisher Scientific). The specificity of fluorescent staining was confirmed by omission of the primary antibody. To quench autofluorescence (lipofuscin), sections were incubated in 0.1% of Sudan Black B for 10 minutes at room temperature and washed with 0.5% of Tx‐PBS for 30 minutes. Sections were coverslipped with Fluoromount^TM^ (Diagnostic BioSystems, Pleasanton, CA). Images were collected using a confocal microscope LSM780 (Carl Zeiss, Jena, Germany) in the Central Research Laboratory of Okayama University Medical School. Alexa Fluor 488, Alexa Fluor 594 or Alexa Fluor 647 was excited with 488, 594 or 633 nm laser beams and observed through 493–560 or 493–630, 595–712 or 638–747 nm emission prism windows, respectively.

### Tau immunoblotting

Frozen brain tissue from the caudate nucleus and putamen of the right hemisphere in two AGD and two PSP cases was available. In addition, frozen tissue from the frontal or temporal cortex in one PSP case, one CBD case and one AD case was examined as disease controls. These samples were used for Western blotting according to methods described previously (14, 48). Brain samples (0.5 g) from patients were individually homogenized in 10 mL of homogenization buffer (HB: 10 mM Tris‐HCl, pH 7.5, containing 0.8 M of NaCl, 1 mM of EGTA and 10% sucrose). Sarkosyl was added to the lysates (final concentration: 2%), which were then incubated for 30 minutes at 37°C and centrifuged at 20 000 g for 10 minutes at 25°C. The supernatant was divided into tubes (each 1.3 mL) and centrifuged at 100 000 g for 20 minutes at 25°C. The pellets were further washed with sterile saline (0.5 mL/tube) and centrifuged at 100 000 g for 20 minutes. The resulting pellets were used as the sarkosyl‐insoluble fraction (ppt). The sarkosyl‐ppt was sonicated in 50 μL (/tube) of 30 mM Tris‐HCl (pH 7.5) and solubilized in 2 × sample buffer. Samples were run on gradient 4–20% polyacrylamide gels and electrophoretically transferred to PVDF membranes. Residual protein‐binding sites were blocked by incubation with 3% of gelatin (Wako) for 10 minutes at 37°C, followed by overnight incubation at room temperature with primary anti‐tau antibodies [T46, mouse, monoclonal, 1:2000. pS396, rabbit, monoclonal, 1:2000 (23, 68)]. The membrane was incubated for 1 h at room temperature with anti‐mouse IgG (BA‐2000, Vector Lab) or anti‐rabbit IgG (BA‐2000, Vector Lab), then incubated for 30 minutes with avidin‐horseradish peroxidase (Vector Lab), and the reaction product was visualized by using 0.1% of DAB and 0.2 mg/mL of NiCl_2_ as the chromogen.

### Statistical analysis

The Mann–Whitney U test and Fisher’s exact test were used to compare the variables between two groups. When comparing the variables between three groups, Kruskal–Wallis and Steel–Dwass tests were used. Spearman rank order correlation analysis was applied for univariate correlations between two variables. To assess the effects of predictor variables on the formation of GFAs, we constructed a multivariate ordered logistic regression model. The stages of GFAs (stages 0 to 4) were categorized into three levels (stage 0, stage 1 and stages 2 to 4) by combining categories in which the number of cases was small, and the data were submitted as the dependent variable in the model. Independent variables tested for their association with the dependent variable consisted of the age at death, Braak stage (stages 0 to 6), Thal phase (phases 0 to 5), PSP (presence or absence) and AGD (presence or absence). Because univariate analyses demonstrated the possibility that clinical and pathological factors may have different effects on GFA formation by anatomical region, the multivariate analysis was carried out after stratification by anatomical region (ie, the frontal cortex, caudate nucleus, putamen and amygdala). Odds ratios (ORs) and 95% confidence intervals (CIs) were calculated after controlling simultaneously for potential confounders. A *P*‐value < 0.01 was accepted as significant. Statistical analysis was performed using Bell Curve for Excel 2.15 (Social Survey Research Information Co., Ltd., Tokyo, Japan).

## Results

### Morphological and immunohistochemical features of GFAs in all subjects

**Figure 2:**
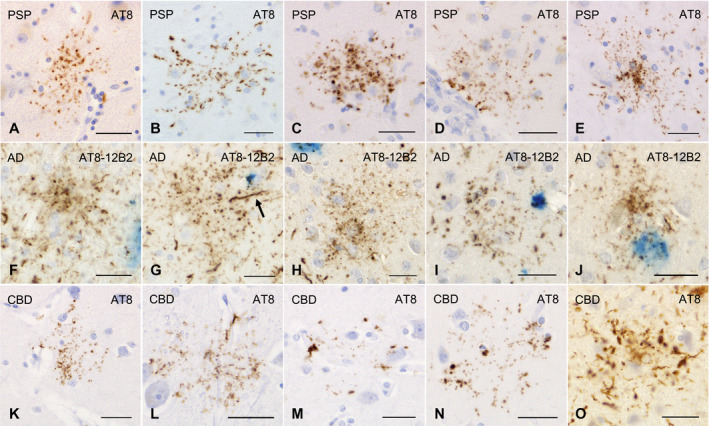
GFAs and disease‐specific astrocytic lesions in PSP, AD and CBD cases. **A**–**E**. GFAs in the amygdala (**A**, **E**), caudate nucleus (**B**, **C**) and putamen (**D**) in PSP cases. AT8 immunohistochemistry. **F**–**J**. Double immunohistochemistry for tau (AT8, blown) and Aβ (12B2, blue) of GFAs in AD cases. The frontal cortex. **F**–**J**. Most GFAs in AD cases did not overlap Aβ‐positive plaques (**F**–**H**). In AD cases, GFAs were often associated with a few short thick dystrophic neurites that may originate from neurons (an arrow) (**G**) and had very fine powdery tau‐positive granules. **K**–**O**. GFAs and astrocytic plaques in CBD cases. AT8 immunohistochemistry. **K**, **L**. GFAs in a CBD case that could not be morphologically differentiated from those in AGD cases. The frontal cortex. **M**, **N**. Astrocytic plaque‐like tau pathologies in a CBD case. Tau tends to be relatively predominantly accumulated in the distal portion of astrocytic processes. The frontal cortex. **O**. Typical astrocytic plaques in a CBD case. The frontal cortex. All scale bars = 30 μm.

**Figure 3:**
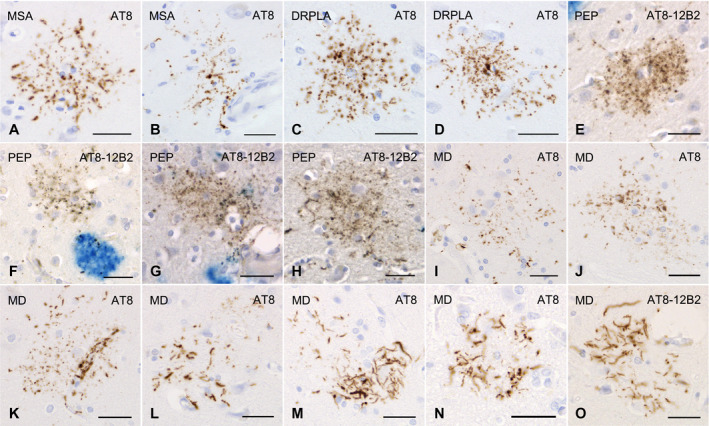
GFAs and other distinctive tau‐positive astrocytic lesions in cases of multiple system atrophy, dentatorubral‐pallidoluysian atrophy, postencephalic parkinsonism and myotonic dystrophy. **A**, **B**. GFAs in an MSA case with PART but without any other primary tauopathy. AT8 immunohistochemistry. The amygdala. **C**, **D**. GFAs in a DRPLA case with PART but without any other primary tauopathy. AT8 immunohistochemistry. The amygdala. **E**–**H**. Double immunohistochemistry for tau (AT8, brown) and Aβ (12B2, blue) of GFA‐like astrocytic tau pathologies in a case of postencephalitic parkinsonism. Glial lesions that are composed of fine tau‐positive granules are usually independent of Aβ deposits. The amygdala. **I**–**O**. GFAs and distinct tau‐positive astrocytic lesions in cases of myotonic dystrophy (MD). AT8 immunohistochemistry. **I**–**K**. GFAs in an MD case. The amygdala. Some GFAs were in contact with vessel walls, in which tau was densely accumulated in astrocytic endfeet touching the vessel (**K**). **L**. Tau‐positive astrocytic lesions having both twisted or crinkly thin threads and granular components like those observed in GFAs in an MD case. The frontal cortex. **M**, **N**. Tau‐positive astrocytic lesions that include almost only twisted or crinkly thin threads in an MD case. The frontal cortex. **O**. Astrocytic lesions mainly composing of longer twisted tau‐positive threads in addition to various quantities of fine tau‐positive granules are often noted in an MD case. Twisted fine threads are often radially arranged, appearing to be reminiscent of threads in astrocytic plaques in CBD. However, the threads of astrocytic lesions in MD cases tend to be finer and longer than those usually observed in astrocytic plaques in CBD cases (see Figure 2O). The frontal cortex. All scale bars = 30 μm.

All the AGD cases had GFAs in at least one region examined (Figure 1A–G). GFAs were composed of small tau‐positive granules that were scattered around astrocytic nuclei or often radially arranged around the nuclei, and often in contact with vessel walls. These lesions were labeled with a series of anti‐tau antibodies except for RD3. Some GFAs were also p62‐positive (Figure 1H). In AGD cases, the immunoreactive pattern of TAs was similar to that of GFAs (Figure 1I–O); however, no GFA having small argyrophilic granules was noted on Gallyas‐stained sections. All PSP cases also had both GFAs and TAs in which immunoreactivity and morphological features were similar to those in AGD cases, respectively (Figure 2A–E). In the frontal cortex in AGD and PSP cases, GFAs tended to be distributed in the ridge of the convolution rather than the depth of the sulci, especially when the number of lesions was small. Nine of 20 AD cases had a small number of GFAs in at least one region (Figure 2F–J). Some GFAs in AD cases were observed to be spatially associated with a few short thick dystrophic neurites that may have originated from neurons (Figure 2G, an arrow). Even in AD cases, the overlap between GFAs and Aβ deposits was rare (Figure 2F–J). Seven of all 18 PART cases had GFAs at least in one region examined (Figure 3A–D).

Of seven CBD cases, one had a small number of GFAs in all regions examined (Figure 2K,L), while all CBD cases had many unclassifiable astrocytic lesions reminiscent of classic astrocytic plaques in which granular, oval or rod‐like tau accumulations tended to be distributed in the peripheral region (Figure 2M,N) in addition to classic astrocytic plaques (Figure 2O). One case of postencephalitic parkinsonism had a few GFAs in the frontal cortex, caudate nucleus and putamen in addition to a larger number of distinct astrocytic lesions having very fine tau‐positive granules (Figure 3E–H). Three of four cases of myotonic dystrophy had a few GFAs in the frontal cortex, caudate nucleus and/or amygdala (Figure 3I,J). All cases of myotonic dystrophy had characteristic tau‐positive astrocytic lesions in which crinkly or twisted thread‐like structures were distributed in the peripheral rather than the central portion (Figure 3K–O). One case with DNTC had a few GFAs in the putamen.

### Frequency and distribution patterns of GFAs and TAs in AGD, PSP, AD and PART groups

**Figure 4:**
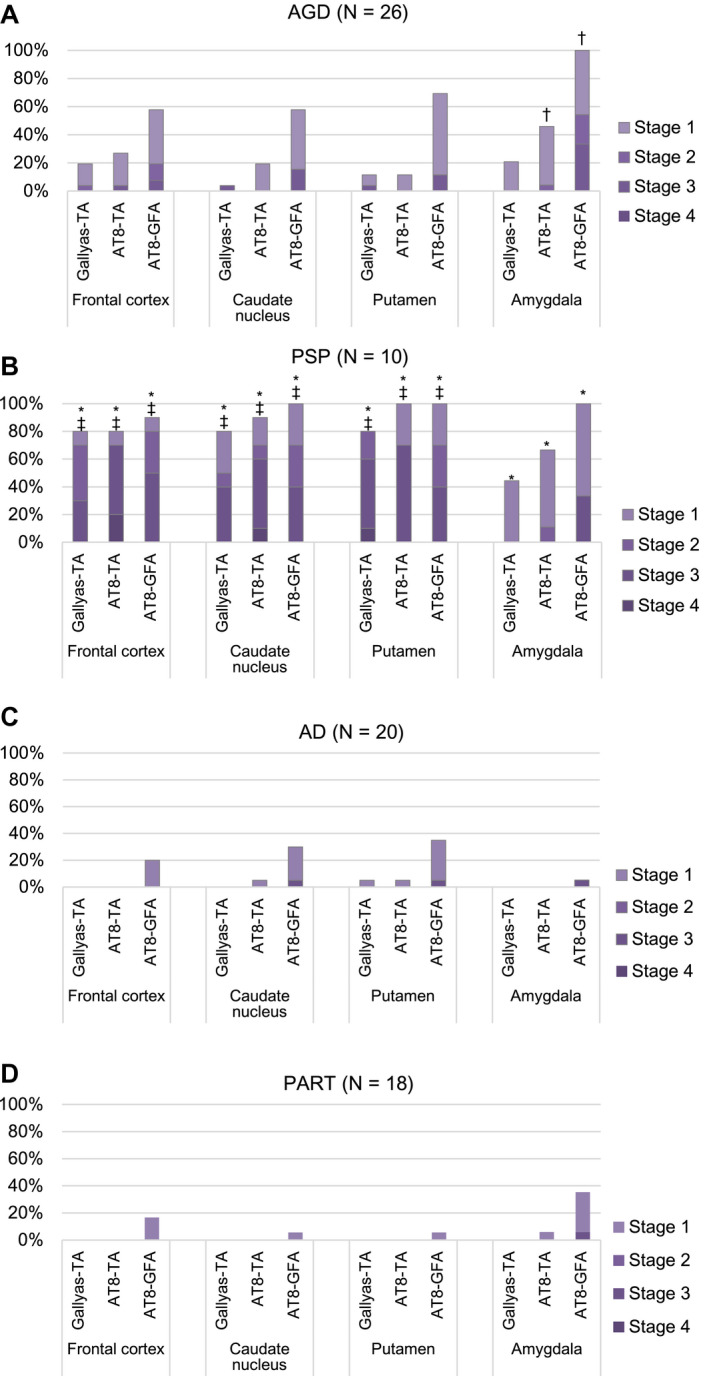
Distribution and density of AT8‐positive GFAs, AT8‐positive TAs and Gallyas‐positive TAs in AGD, PSP, AD and PART groups. **A**. In AGD cases (N = 26), GFAs and TAs show similar distributions: both lesions most frequently develop in the amygdala, and GFAs are more frequent that TAs in all regions examined. **B**. In PSP cases (N = 10), GFAs were almost consistently noted in all regions examined, and the proportions of cases having the high density GFAs (stage 2 or over) in the striatum and frontal cortex were higher than that in the amygdala. The distribution pattern of TAs was similar to that of GFAs. **C**. In AD cases (N = 20), GFAs were more frequent in the striatum compared with the frontal cortex and amygdala, although less frequent than in AGD and PSP cases. **D**. In PART cases (N = 18), GFAs were the most frequent in the amygdala, whose distribution pattern was similar to that in an AGD rather than AD and PSP groups. *Significant differences between PSP and AD groups (*P* < 0.01). †Significant differences between AGD and AD groups (*P* < 0.01). ‡Significant differences between AGD and PSP groups (*P* < 0.01). Kruskal–Wallis and Steel–Dwass tests.

AGD cases, PSP cases without AGD, AD cases without AGD and PART cases without AGD showed distinct distribution patterns of GFAs (Figure 4A–D). In AGD cases, GFAs were consistently noted in the amygdala (100.0%), followed by the putamen (69.2%) and caudate nucleus and frontal cortex (57.7%, respectively) and the high density of GFAs of stage 2 or over was frequently found in the amygdala (54.2%) rather than the frontal cortex, caudate nucleus and putamen (19.2%, 15.4% and 11.5%, respectively) (Figure 4A). In PSP cases without AGD, in contrast to AGD cases, a large number of GFAs were consistently noted in the amygdala, caudate nucleus and putamen (100.0%, respectively), and also frequently in the frontal cortex (90.0%) (Figure 4B), and the high density of GFAs of stage 2 or over was frequently found in the frontal cortex, caudate nucleus, putamen (80%, 70% and 70%, respectively) rather than the amygdala (33.3%) (Figure 4B). In AD cases without AGD, although far less frequent compared with AGD and PSP groups, GFAs were noted in the putamen (35.0%), followed by the caudate nucleus (30.0%), frontal cortex (20.0%) and amygdala (5.3%) (Figure 4C). In PART cases without AGD, GFAs were most frequently noted in the amygdala (35.3%), followed by the frontal cortex (16.7%) and the caudate nucleus and putamen (5.6%, respectively), being similar to the distribution pattern observed in AGD rather than AD cases (Figure 4D). The age at death, sex, brain weight, Braak stage, Thal phase, the proportion of cases with Lewy body disease (LBD) and the proportion of cases with TDP‐43 pathology were not significantly different between PART cases with and without GFAs (Mann–Whitney U test and Fisher’s exact test; Supporting File S1).

In AGD, PSP, AD and PART groups, the distribution pattern of GFAs was similar to those of AT8‐positive TAs and Gallyas‐positive TAs, respectively (Figure 4A–D).

In the comparison between AGD, PSP and AD groups, the densities of GFAs in the amygdala in an AGD group were significantly larger than those in the AD group, respectively (*P* < 0.001, Kruskal–Wallis and Steel–Dwass tests). The densities of GFAs in all regions in the PSP group were significantly larger than those in the AD group (*P* < 0.001, respectively, Kruskal–Wallis and Steel–Dwass tests). The densities of GFAs in the frontal cortex, caudate nucleus and putamen in the PSP group were significantly larger than those in the AGD group (*P* = 0.006, 0.007 and 0.004, respectively, Kruskal–Wallis and Steel–Dwass tests).

According to recently proposed distribution patterns of ARTAG (44), the distributions of GFAs in AGD and PART cases were similar to pattern 1 (amygdala first pattern), while the distribution of GFAs in AD cases was similar to pattern 2 (striatum first pattern), respectively (Supporting File S2).

### Age at death and pathological features by region in all cases with or without GFAs

We then compared the age at death and pathological features of all cases with and without GFAs to explore factors potentially associated with GFA formation (Table 3). The age at death in cases having GFAs in the caudate nucleus and putamen was significantly higher than that in cases lacking GFAs, respectively (*P* = 0.005 and <0.001, respectively, Mann–Whitney U test; Table 3). The AGD stage in cases having GFAs in the caudate nucleus, putamen and amygdala was significantly higher than that in cases lacking GFAs (*P* = 0.004, 0.002, and <0.001, respectively, Mann–Whitney U test). The frequency of PSP cases was significantly higher in cases having GFAs than in cases lacking GFAs in all regions, respectively (*P* < 0.001, respectively, Fisher’s exact test). Braak stage, Thal phase and the frequencies of LBD and TDP‐43‐positive lesions did not differ between the two groups.

**Table 3:** Demographic and pathological features of all cases with or without GFAs

	Frontal cortex (N = 105)		Caudate nucleus (N = 105)		Putamen (N = 105)		Amygdala (N = 96)	
	Cases with GFAs	Cases without GFAs	Cases with GFAs	Cases without GFAs	Cases with GFAs	Cases without GFAs	Cases with GFAs	Cases without GFAs
N (%)	36 (34.3)	69 (65.7)	35 (33.3)	70 (66.7)	39 (37.1)	66 (62.9)	43 (44.8)	53 (55.2)
Age at death [y, mean ± SD]^†^	74.8 ± 11.5	69.2 ± 12.2	75.7 ± 10.5*	68.8 ± 12.4	77.2 ± 9.6*	67.5 ± 12.2	73.7 ± 10.3	69.2 ± 13.5
Braak stage [median (25^th^ percentile, 75^th^ percentile)]^†^	2 (2, 4)	2 (1, 5)	3 (2, 4)	2 (1, 5)	3 (2, 4)	2 (1, 4.75)	2 (2, 4)	3 (1, 5)
Thal phase [median (25^th^ percentile, 75^th^ percentile)]^†^	1 (0, 3)	0 (0, 3)	1 (0, 3)	0 (0, 3)	1 (0, 3)	0 (0, 3)	1 (0, 3)	1 (0, 4)
AGD stage [median (25^th^ percentile, 75^th^ percentile)]^†^	0 (0, 2)	0 (0, 0)	0 (0, 2)*	0 (0, 0)	0 (0, 2)*	0 (0, 0)	1 (0, 2)*	0 (0, 0)
PSP (N, %)^‡^	9 (90.0)*	1 (10.0)	10 (100.0)*	0 (0.0)	10 (100.0)*	0 (0.0)	9 (100.0)*	0 (0.0)
CBD (N, %)^‡^	1 (14.3)	6 (85.7)	1 (14.3)	6 (85.7)	1 (14.3)	6 (85.7)	1 (16.7)	5 (83.3)
LBD (N, %)^‡^	7 (28.0)	18 (72.0)	9 (36.0)	16 (64.0)	9 (36.0)	16 (64.0)	7 (30.4)	16 (69.6)
TDP‐43 pathology (N, %)^‡^	11 (32.4)	23 (67.6)	14 (41.2)	20 (58.8)	14 (41.2)	20 (58.8)	12 (35.3)	22 (64.7)
PART only (N, %)^‡^, ^§^	2 (40.0)	3 (60.0)	0 (0.0)	5 (100.0)	1 (20.0)	4 (80.0)	3 (75.0)	1 (25.0)

Abbreviations: AGD = argyrophilic grain disease; CBD = corticobasal degeneration; GFA = granular/fuzzy astrocyte; LBD = Lewy body disease; PSP = progressive supranuclear palsy; TDP‐43 pathology = all cases having FTLD‐TDP, ALS‐TDP and other TDP‐43‐positive lesions in the limbic system and neocortex are included.

* *P* < 0.01.

^†^ Mann–Whitney U test.

^‡^ Frequency of each lesion was compared using Fisher’s exact test.

^§^ PART cases without any other tauopathies and non‐tauopathies. The amygdala was not available in one of these PART cases.

### Correlation analyses of GFA stage and clinical and pathological variables by region in all subjects

The correlations between the GFA stage and clinical and pathological variables in all subjects were analyzed using Spearman rank order correlations (Supporting File S3). The AGD stage was significantly correlated with the GFA stage in the putamen and amygdala, respectively (*ρ* = 0.271 and 0.530; *P* = 0.005 and <0.001). On the contrary, the age at death was significantly correlated with the GFA stage only in the caudate nucleus and putamen (*ρ* = 0.267 and 0.358; *P* = 0.006 and <0.001).

The GFA stages in the frontal cortex, caudate nucleus, putamen and amygdala were significantly correlated with the density of Gallyas‐positive TAs in the same regions (*ρ* = 0.647, 0.507, 0.534 and 0.332; *P* < 0.001, respectively). Neither Braak stage nor Thal phase was significantly correlated with the GFA stage in any region.

### Effects of age, Braak stage, Thal phase, PSP and AGD on GFA formation by region

Ordered logistic regression analyses were used to examine whether the age at death, Braak stage, Thal phase, presence or absence of PSP and presence or absence of AGD were independently associated with the increase of GFA stage by region in all cases (Table 4).

**Table 4:** Ordered logistic regression analyses of associations between density of GFAs, age, Braak stage, Thal phase, PSP and AGD by anatomical region in all subjects

	Odds ratio	95% confidence interval	*P*‐value
*GFAs in the frontal cortex*			
Age at death	1.03	0.98–1.08	0.325
Braak stage	1.27	0.90–1.79	0.171
Thal phase	0.87	0.63–1.20	0.391
PSP	214.19	29.16–1573.46	<0.001*
AGD	7.30	2.31–23.04	<0.001*
*GFAs in the caudate nucleus*			
Age at death	1.02	0.96–1.07	0.582
Braak stage	1.54	1.02–2.32	0.038
Thal phase	1.07	0.77–1.49	0.686
PSP	483.92	56.76–4125.91	<0.001*
AGD	14.68	3.69–58.41	<0.001*
*GFAs in the putamen*			
Age at death	1.07	1.01–1.13	0.022
Braak stage	1.67	1.10–2.54	0.015
Thal phase	0.84	0.60–1.17	0.293
PSP	1020.11	96.40–10795.50	<0.001*
AGD	15.66	3.98–61.65	<0.001*
*GFAs in the amygdala*			
Age at death	1.00	0.95–1.06	0.947
Braak stage	1.14	0.81–1.61	0.454
Thal phase	0.73	0.51–1.05	0.093
PSP	52.95	9.32–300.92	<0.001*
AGD	118.14	23.48–594.39	<0.001*

* *P* < 0.01.

Abbreviations: AGD = the presence of AGD lacking any other primary tauopathies; GFA = granular/fuzzy astrocyte; PSP = the presence of PSP lacking any other primary tauopathies.

In the frontal cortex, the strongest independent factor of GFA formation was the pathological diagnosis of PSP (OR 214.19; 95% CI 29.16–1573.46), followed by the presence of AGD (OR 7.30; 95% CI 2.31–23.04). Likewise, in the caudate nucleus, PSP (OR 483.92; 95% CI 56.76–4125.91) was the strongest factor associated with GFA formation, followed by AGD (OR 14.68; 95% CI 3.69–58.41). In the putamen also, the strongest factor of GFA formation was PSP (OR 1020.11; 95% CI 96.4–10795.5), followed by AGD (OR 15.66; 95% CI 3.98–61.65).

In contrast, in the amygdala, the pathological diagnosis of AGD had the strongest association with GFA formation (OR 118.14; 95% CI 23.5–594.4), followed by PSP (OR 52.95; 95% CI 9.3–300.9). The age at death, Braak stage or Thal phase was not significantly associated with GFA formation in any region examined.

### Relationship between change of immunoreactivity of GFAs and TAs and progression of argyrophilic grains in AGD cases

**Figure 5:**
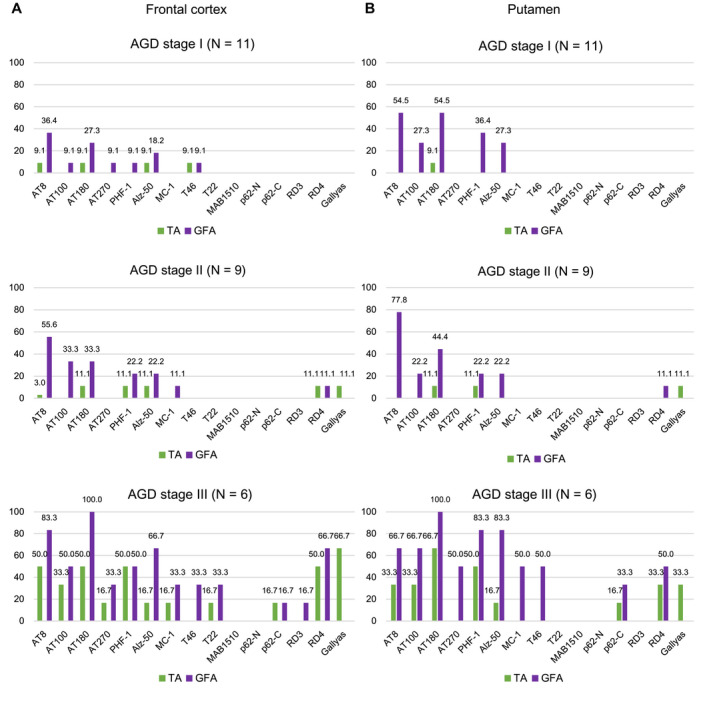
Changes of immunoreactivity of GFAs and TAs parallel the progression of the AGD stage in the frontal cortex (**A**) and putamen (**B**) in AGD cases. About 35% of cases with AGD stage I had GFAs labeled by several anti‐tau antibodies in the frontal cortex and putamen. Neither Gallyas‐ nor p62‐positive lesions were noted in these cases. Gallyas‐positive TAs were first noted in cases with AGD stage II, and the proportion of AGD cases with TAs increased with AGD stage. p62‐positive GFAs were first noted in cases with AGD stage III. In this study, when an astrocytic lesion had Gallyas‐positive glial threads, we operationally regarded it as a TA irrespective of the quantity of Gallyas‐positive threads.

Based on the significant correlation between the stages of GFAs and AGD in all regions demonstrated by univariate and multivariate analyses (Table 4 and Supporting File S3), to explore findings associated with the formation process of GFAs, the change of immunoreactivity of GFAs in the frontal cortex and putamen in AGD cases was examined using a panel of anti‐tau, ubiquitin and p62 antibodies and the Gallyas method after stratification by AGD stage (Figure 5A,B). In this examination, astrocytic lesions having Gallyas‐positive glial threads were regarded as TAs regardless of the quantity of argyrophilic structures. In both regions examined, with the progression of AGD stage, phosphorylation‐ and conformation‐dependent anti‐tau antibodies increasingly labeled GFAs and TAs, respectively (Figure 5A,B). Gallyas‐positive TAs were noted only in cases with AGD stages II or higher. p62‐positive GFAs occurred only in cases with AGD stage III.

### Double staining with AT8 immunohistochemistry and Gallyas method

**Figure 6:**
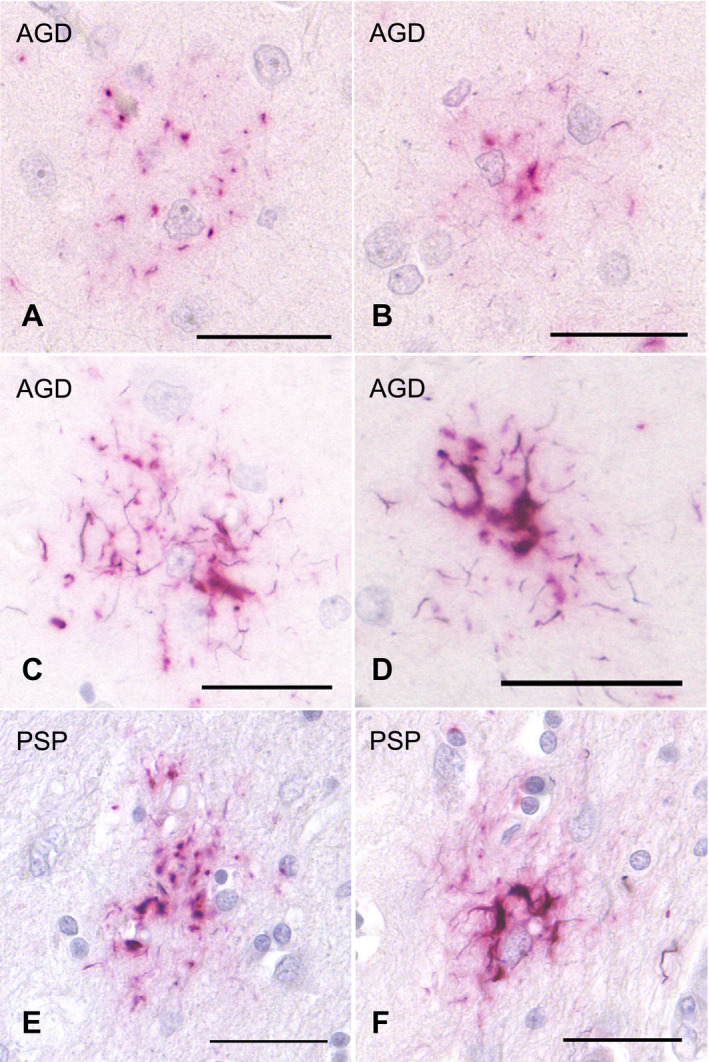
Double staining with AT8 immunohistochemistry and Gallyas method of tau‐positive astrocytic lesions in AGD and PSP cases. **A**, **B**. Some GFA‐like astrocytic lesions having various quantities of fine granular tau accumulation and a few Gallyas‐positive threads in an AGD case. The frontal cortex. **C**, **D**. TAs having Gallyas‐positive threads in AGD cases. The frontal cortex. **E**, **F**. GFA‐like astrocytic lesion (**E**) and TA (**F**) having various quantities of tau‐positive granules and Gallyas‐positive threads in PSP cases. The putamen. All scale bars = 30 μm.

Double staining with AT8 immunohistochemistry and the Gallyas method demonstrated astrocytic lesions having fine GFA‐like granular tau accumulation, and like TAs, a few to a moderate number of Gallyas‐positive glial threads in AGD cases (Figure 6A–D). Some lesions had thicker Gallyas‐positive threads (Figure 6D), being indistinguishable from TAs in PSP cases (Figure 6F). Similar astrocytic lesions having both fine tau‐positive granules and Gallyas‐positive threads were noted in PSP cases also (Figure 6E).

### Confocal laser scanning microscopy

**Figure 7:**
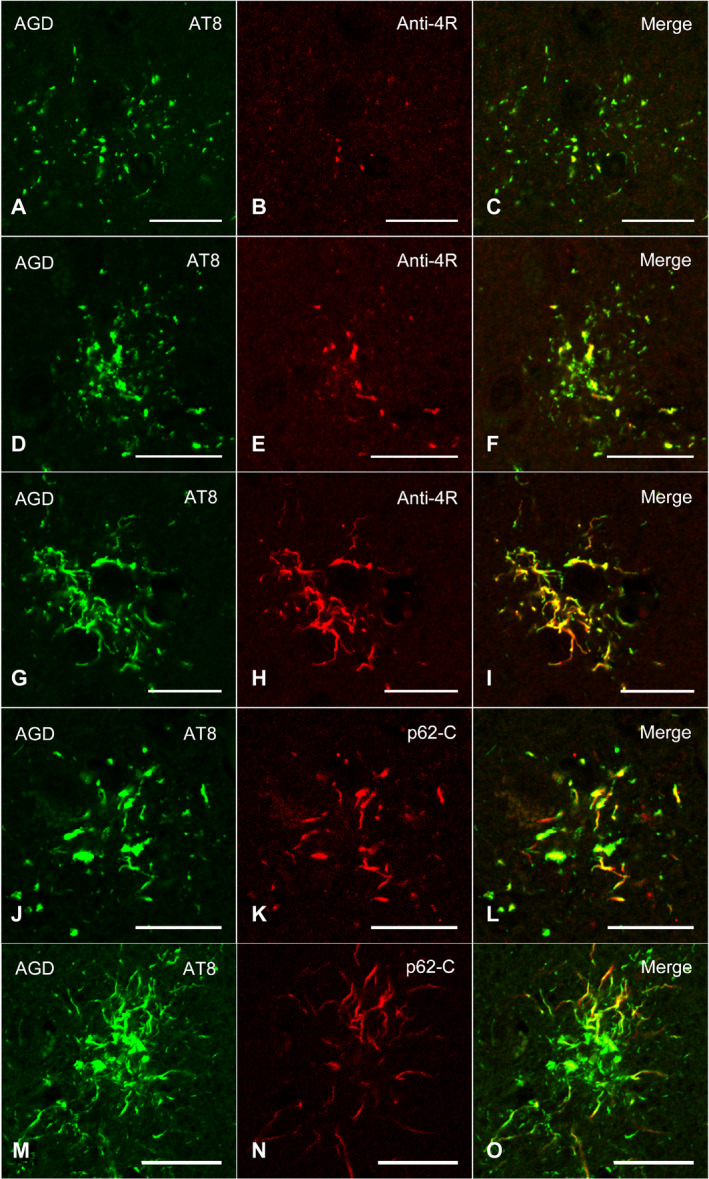
Confocal double‐immunofluorescence of the combination of AT8 (**A**, **D**, **G**) and anti‐4R tau antibodies (**B**, **E**, **H**), and the combination of AT8 (**J**, **M**) and p62‐C (**K**, **N**) in the frontal cortex in an AGD case. **A**–**C**, **D**–**F**. GFAs. An AT8 epitope is partially colocalized with an anti‐4R tau epitope. **G**–**I**. A TA. An anti‐4R tau epitope is often colocalized with an AT8 epitope. **J**–**L**. A GFA. Some GFAs having not only fine tau granules, but also coarse tau granules showed the colocalization of epitopes of AT8 and p62‐C. **M**–**O**. A TA. Some TAs also rarely showed the colocalization of epitopes of AT8 and p62‐C. **C**, **F**, **I**, **L**, **O**. Merged images. All scale bars = 20 μm.

In AGD cases, double labeling with AT8 and anti‐4R tau antibodies showed the accumulation of 4R tau in GFAs (Figure 7A–F). Some TAs were intensely stained with anti‐4R tau antibody in AGD cases (Figure 7G–I). The accumulation of p62 was revealed in AT8‐positive GFAs and TAs with relatively thick glial threads (Figure 7J–O).

### Tau immunoblotting in representative AGD and PSP cases

**Figure 8:**
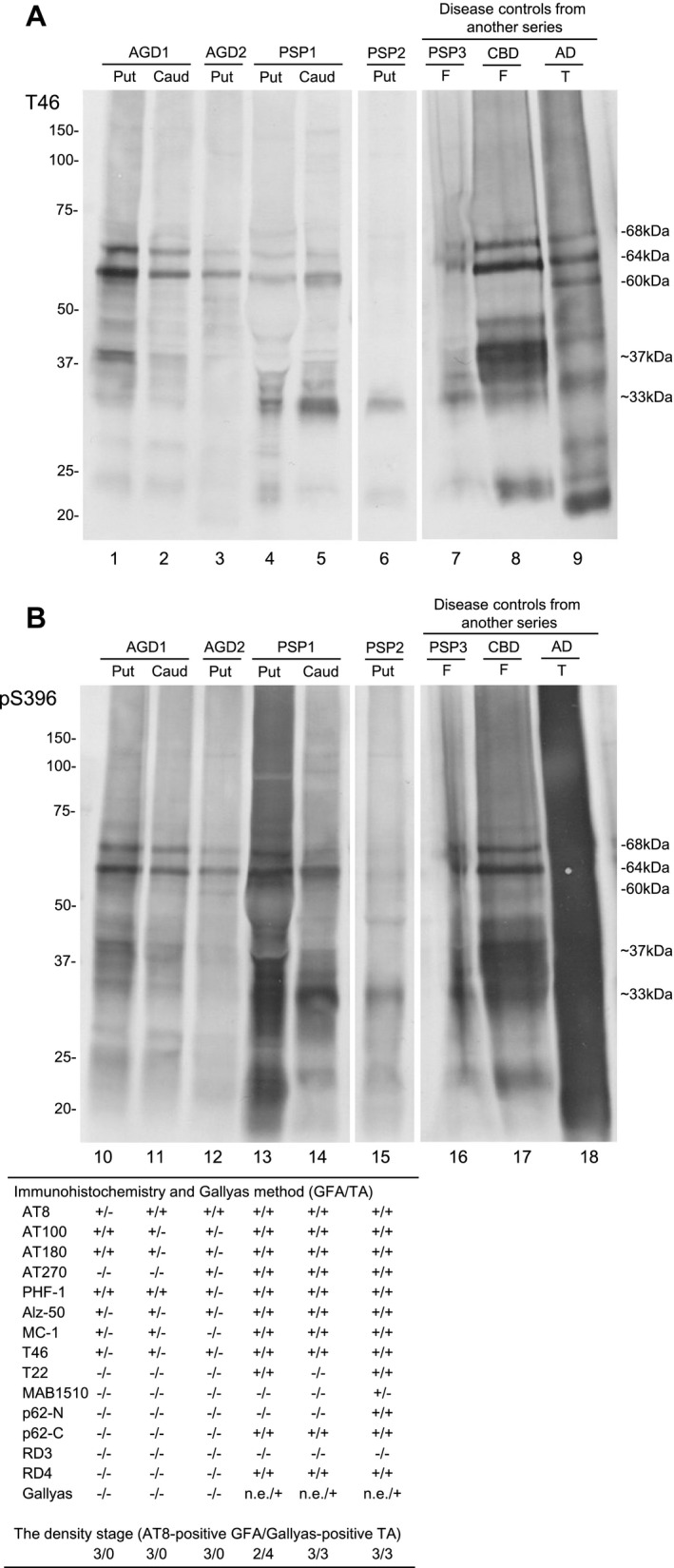
Tau immunoblot analysis of the sarkosyl‐insoluble, urea‐soluble fraction prepared from the right hemisphere of representative AGD (AGD1 and AGD2), PSP (PSP1 and PSP2) and disease control cases (PSP3, CBD and AD) with T46 (**A**) and pS396 (**B**). All bands and smears tended to be more strongly stained with pS396 rather than T46. Approximately 68‐ and 64‐kDa bands are seen in all AGD (lanes 1–3, 10–12), PSP (lanes 4–7, 13–16) and CBD cases (lanes 8 and 17). Cases of PSP (lanes 4–6, 13–15) and CBD (lanes 8 and 17) show 33‐ and 37‐kDa mass tau fragments, respectively. One of two AGD cases showed less intensely stained 33‐ to 37‐kDa tau fragments in the putamen and caudate nucleus (lanes 1, 2, 10 and 11). In the other AGD case, the immunoreactivity of 33‐ to 37‐kDa tau fragments in the putamen was very weak (lanes 3 and 12). On tau immunohistochemistry in corresponding regions of the left hemisphere, the density of GFAs were comparable between AGD and PSP cases (Table of Figure 8B). However, TAs tended to be stained with a larger number of anti‐tau antibodies in PSP cases than AGD cases. Further, in these cases, GFAs and TAs stained with anti‐p62 antibodies and/or Gallyas method were noted only in PSP cases. Abbreviations: AGD = argyrophilic grain disease; AD = Alzheimer’s disease; Caud = caudate nucleus; CBD = corticobasal degeneration; F = frontal cortex; PSP = progressive supranuclear palsy; Put = putamen; n.e. = Gallyas‐positive GFAs were not evaluated in PSP cases because many Gallyas‐positive dot‐like structures were scattered, and thereby made it difficult to count Gallyas‐positive GFAs; T = temporal cortex.

Immunoblot analysis of the sarkosyl‐insoluble, urea‐soluble fraction prepared from the right hemisphere with T46 and pS396 demonstrated approximately 68‐ and 64‐kDa bands in all AGD, PSP and CBD cases (Figure 8A, lanes 1–8 and Figure 8B, lanes 10–17). All bands and smears tended to be more strongly stained with pS396 than T46. Low molecular weight tau fragments of approximately 33‐ and 37‐kDa were also noted in PSP (lanes 4–7 and 13–16) and CBD (lanes 8 and 17), respectively. Of two AGD cases, one case (AGD1) showed approximately 33‐ to 37‐kDa tau fragments in the putamen and caudate nucleus (lanes 1, 2, 10 and 11). The immunointensity of the fragments was less than those in PSP cases. The other AGD case (AGD2) showed very weakly stained 33‐ to 37‐kDa tau fragments in the putamen (lanes 3 and 12). The density of GFAs in corresponding regions in the left hemisphere was comparable between these AGD and PSP cases (table of Figure 8B). On the contrary, GFAs and TAs tended to be stained with a larger number of anti‐tau antibodies in PSP cases (lanes 13–15) than AGD cases (lanes 10–12). In these cases, GFAs and TAs stained with anti‐p62 antibodies and/or Gallyas method were noted only in PSP cases (lanes 13–15).

## Discussion

This is the first study that demonstrated distinct distributions of GFAs in AGD, PSP, AD and PART cases and comprehensively evaluated the impacts of patient age and major pathological factors on GFA formation by anatomical region. In AGD cases, GFAs were consistently noted in the amygdala, and to a lesser frequency, in the striatum and frontal cortex (57–69%). In PSP cases, GFAs were almost consistently noted in all regions, and the proportion of cases having the high density of GFAs was higher in the striatum and frontal cortex than in the amygdala. In contrast, only 35% of AD cases showed a few GFAs in the striatum, followed by the frontal cortex (20%) and amygdala (5%). Although the PART cases lacked AGD or any other primary tauopathies, 35% had a few GFAs with a distribution more similar to that in AGD than in AD cases. In multivariate ordered logistic regression analyses, PSP was the strongest independent factor of GFA formation in the frontal cortex, caudate nucleus and putamen, while AGD was the strongest independent factor of GFA formation in the amygdala. In contrast, neither age nor AD pathology was significantly associated with GFA formation in any region examined. In AGD cases, GFAs labeled with phosphorylation‐ and conformation‐dependent anti‐tau antibodies, Gallyas‐positive glial threads in GFAs and the activation of autophagy sequentially occurred parallel to the progression of AGD. These findings suggest that GFAs may develop with a distinct distribution pattern associated with underlying tauopathies rather than age.

Cases of primary tauopathies often had not only disease‐specific astrocytic lesions, but also not negligible numbers of morphologically unclassifiable tau‐positive astrocytic lesions (39, 43, 44). Indeed, in our cases of PSP, CBD, myotonic dystrophy and postencephalitic parkinsonism, unclassifiable tau‐positive astrocytic lesions having both morphological features of GFAs and disease‐specific astrocytic lesions were observed. Therefore, it is considered that where the boundary in the morphological evaluation is set may affect the frequency of GFAs in each study. In this study, to secure the reproducibility of results, only astrocytic lesions which showed morphological features of classic GFAs originally reported in AGD cases were regarded as GFAs (4). The previously reported higher frequency of GFAs in CBD cases (e.g., about 90% in the frontal cortex and striatum in the CBD cases (44) vs. 14.3% in the sites in our CBD cases) may be at least partially explained by such methodological issues, it being difficult to simply compare the frequency of GFAs between studies.

Although there are several previous studies regarding the immunoreactivity of GFAs (17, 45, 60), the change of immunoreactivity related to the process of GFA formation is hardly known. In our study, because the AGD stage was significantly correlated with the density of GFAs (Table 4), we considered that the change of immunohistochemical features of GFAs associated with the progression of the AGD stage may reflect the formation process of GFAs, at least in AGD cases. We selected the frontal cortex and putamen as regions of interest for this examination because the number of GFAs in these sites varied between cases. As a result, it was demonstrated that the increase of GFAs labeled with phosphorylation‐ and conformation‐dependent anti‐tau antibodies and the activation of autophagy occurred in the regions parallel to the progression of AGD (Figure 5). It was also noteworthy that, with the increase of GFAs, Gallyas‐positive glial threads indistinguishable from those observed in TAs in PSP cases gradually increased in GFAs in AGD cases (Figure 5), and that the distribution of TAs was consistent with that of GFAs (Figure 4). Further, Gallyas‐AT8 double staining demonstrated that some GFAs in AGD cases actually had a few fine Gallyas‐positive threads in addition to Gallyas‐negative tau‐positive fine granular structures as features of GFAs. Based on these findings, it is plausible that at least some GFAs may precede changes of full‐blown Gallyas‐positive TAs. On the contrary, at present, TAs are considered to be disease‐specific lesions of PSP. Therefore, regardless of the quantity, the presence of TAs is often regarded as an early change of PSP. In this study, Gallyas‐positive TAs were observed in about 40% of AGD cases as well as all PSP cases. However, because the distribution pattern of TAs, like that of GFAs, evidently differs between PSP and AGD cases (Figure 4), we consider that whether a small number of Gallyas‐positive TAs observed in AGD cases always means the coexisting early change of PSP remains to be elucidated. Interestingly, in our results of tau immunoblotting, although the number of cases examined was small, the band pattern of approximately 33‐ to 37‐kDa low‐molecular weight tau fragments might differ between AGD and PSP cases even when the same anatomical region (ie, striatum) was examined (Figure 8). It is also unclear whether the inconsistency reflects distinct pathophysiologies in AGD and PSP, respectively, or reflects a continuum of GFAs and TAs that is shared by two tauopathies. Further comparative studies between immunohistochemical and biochemical findings using a larger number of cases are necessary to clarify the pathogenic significances of GFAs and TAs in tauopathies, especially in AGD and PSP.

Kovacs *et al* (44) statistically explored potential progression patterns of ARTAG in a large case series including cases of CBD, PSP, Pick’s disease, PART, AD, MSA, LBD and ALS. AGD coexisted in some cases, although detailed information, for example, the number of cases having AGD and the severity of AGD, was not shown. In their study, they statistically extracted two potential progression patterns of GFAs, that is, patterns 1 and 2 (44). In pattern 1, GFAs may first develop in the striatum and extend to the frontotemporal cortex [the striatum first pattern (37)]. Interestingly, this pattern appears to be consistent with that of GFAs observed in our AD cases (Figure 4C). Considering that Kovacs’s 528 non‐FTLD tauopathy cases included 243 AD cases (46.0%), it is plausible that pattern 1 mainly reflects the distribution of GFAs in their AD cases. Regarding the distribution of GFAs in AD cases, a recent study examined the frequency of GFAs in the frontal, temporal, cingulate, angular gyri, putamen, amygdala and entorhinal cortex (59). Although GFAs were comparable in density in these regions, in their study, unlike ours, AD cases with AGD were not excluded because of their high frequency. However, given our findings, such a selection might increase the frequency of GFAs in the amygdala in AD cases. In addition, multivariate analyses demonstrated that AGD was not associated with the presence of GFAs, being inconsistent with our results. However, the effect of AGD on GFA formation was not evaluated by anatomical region. Therefore, their findings cannot be compared with our results. On the contrary, in pattern 2 reported by Kovacs *et al* (44), GFAs may first develop in the amygdala and subsequently in the subcortical nuclei and frontotemporal cortex [the amygdala first pattern (37)], being consistent with the distribution pattern of GFAs observed in our AGD cases (Figure 4A). Somewhat unexpectedly, previously reported findings regarding the detailed distribution and frequency of GFAs in AGD cases are limited. In a previous study including 54 cases of degenerative diseases with coexisting AGD, it was demonstrated that GFAs were most frequent in the medial temporal region including the hippocampus, amygdala and inferior temporal gyrus, and the proportion of cases having GFAs at least in one of these regions was 90.4% (43). In that study, only three cases of AGD without any other primary tauopathy were examined; of these, one case had GFAs at least in one of the regions, although the detailed distribution was not described. In another study, only the effect of grains as coexisting lesions in the hippocampus pyramidal layers, dentate gyrus, inferior temporal gyrus and/or amygdala on GFA formation in the amygdala in a case series of diverse degenerative diseases was examined, and a significant relationship between grains in these regions and GFAs in the amygdala was noted (44). Based on these previous findings together with our results, when GFAs are predominantly distributed in the amygdala, the possibility should be considered that the case may have the pathogenic process associated with the formation of argyrophilic grains.

It is noteworthy that about 35% of our PART cases had GFAs, the distribution of which was consistent with that in AGD cases, although they lacked argyrophilic grains. In a previously reported series of PART cases as well, a similar distribution pattern of GFAs with the highest frequency in the amygdala (29%) was observed (44). These results led us to consider the possibility that at least some PART cases with amygdala‐predominant GFAs actually have not only the pathological process of PART, but also the very early glial change of AGD, even when lacking argyrophilic grains on sections stained with the Gallyas method. This view may be supported by previous statistical analyses using the conditional probability that demonstrated that GFA formation may precede grain formation in the amygdala (44). The distribution pattern of GFAs was evidently different between our AD and PART cases, suggesting that the tau accumulation in astrocytes observed in PART cases may not be an early process of GFA formation in AD. Further, considering that GFAs in the amygdala were rare even in AD cases with a high Braak NFT stage (V–VI), it is unlikely that amygdala GFAs with high frequency in PART cases developed in association with PART with a lower Braak NFT stage (I–IV). On the contrary, our results also appear to suggest that the presence or absence of GFAs in the amygdala may be related to the progression from PART to full‐blown AD pathology. Considering that extensive tau accumulation in the neocortex may require sufficient cortical Aβ accumulation in AD cases (65) and the possibility that amygdala GFAs may be associated with argyrophilic grains (44), the potential relationship can be at least partially explained by the poverty of Aβ deposits and a related genetic background in AGD cases. For example, it has been reported that the frequency of the ApoE ε4 allele in AGD cases was significantly lower than that in AD cases (73), the frequency of the ApoE ε2 allele in AGD cases was significantly higher than that in normal controls (19) and the neocortical Aβ load in AGD cases was significantly lower than that in AD cases (70, 72). These findings may be consistent with the relationship between the lack of amygdala GFAs and the evolution from PART to AD observed in our study. Given these findings, it is plausible that the pathogenic background in PART cases may be heterogeneous, and a genetic background associated with amygdala GFAs should be explored.

Limitations of this study have to be considered. First, a significant factor affecting the results is the small number of cases, especially those of PSP and other rare tauopathies and non‐tauopathies, although a *P*‐value <0.01 was accepted as significant to interpret the results with caution. Second, in our semiquantitative assessment, to strictly set the morphological criteria of GFAs, we counted lesions as GFAs only when they showed the morphological features of GFAs common in the AGD cases reported originally by Botez *et al* (4). In the consensus paper also, the astrocytic lesions observed in AGD brains are regarded as most typical for GFAs (38). The narrow definition of GFAs used in this study might reduce the frequency of lesions. However, considering that the characteristic distribution patterns of GFAs were revealed in several diseases using such an evaluation procedure, focusing on GFAs showing morphological features commonly observed in AGD brains might be rather useful to explore the pathological process in tauopathies. In this context, the pathological relationship between GFAs and thorn‐shaped astrocytes might be also needed to be examined. Third, we presented results of tau immunoblotting using tissues from the right hemisphere with findings of immunoreactivity and argyrophilia of GFAs and TAs in corresponding regions in the left hemisphere in each case. However, the possibility that there might be a discrepancy between the hemispheres regarding tau pathology cannot be denied. Therefore, tau immunohistochemistry and immunoblotting using tissue from the ipsilateral hemisphere in a larger number of AGD cases might provide more suggestive findings to address these issues.

In conclusion, we demonstrated that the development of GFAs may be distinctively affected by underlying pathological bases by region rather than age; therefore, GFAs showed characteristic distribution patterns associated with underlying tauopathies. Whether evaluating the distribution of GFAs is useful to predict underlying tauopathies, especially in the early phase of the pathological process in some diseases, should be further examined. In addition, the potential impact of GFAs on motor and cognitive functions and the psychiatric condition of people with and without neurological diseases may be of clinical interest and should be explored in the future.

## Conflict of Interest

The authors declare that they have no conflict of interest.

## Supporting information

File S1
**Supporting File S1.** Demographic data of 18 PART cases with and without GFAs.

File S2
**Supporting File S2.** Distribution patterns of GFAs in AGD, PSP, AD, and PART cases.

File S3
**Supporting File S3.** Correlation of all cases.

## Data Availability

Research data are not shared.

## References

[1] Ahmed Z , Bigio EH , Budka H , Dickson DW , Ferrer I , Ghetti B *et al* (2013) Globular glial tauopathies (GGT): consensus recommendations. Acta Neuropathol 126:537–544. 23995422 10.1007/s00401-013-1171-0 PMC3914659

[2] Arai T , Hasegawa M , Akiyama H , Ikeda K , Nonaka T , Mori H *et al* (2006) TDP‐43 is a component of ubiquitin‐positive tau‐negative inclusions in frontotemporal lobar degeneration and amyotrophic lateral sclerosis. Biochem Biophys Res Commun 351:602–611. 17084815 10.1016/j.bbrc.2006.10.093

[3] Armstrong RA , Lantos PL , Cairns NJ (2007) Progressive supranuclear palsy (PSP): a quantitative study of the pathological changes in cortical and subcortical regions of eight cases. J Neural Transm 114:1569–1577. 17680229 10.1007/s00702-007-0796-3

[4] Botez G , Probst A , Ipsen S , Tolnay M (1999) Astrocytes expressing hyperphosphorylated tau protein without glial fibrillary tangles in argyrophilic grain disease. Acta Neuropathol 98:251–256. 10483782 10.1007/s004010051077

[5] Bowser R , Giambrone A , Davies P (1995) FAC1, a novel gene identified with the monoclonal antibody Alz50, is developmentally regulated in human brain. Dev Neurosci 17:20–37. 7621746 10.1159/000111270

[6] Braak H , Alafuzoff I , Arzberger T , Kretzschmar H , Del Tredici K (2006) Staging of Alzheimer disease‐associated neurofibrillary pathology using paraffin sections and immunocytochemistry. Acta Neuropathol 112:389–404. 16906426 10.1007/s00401-006-0127-z PMC3906709

[7] Braak H , Braak E (1987) Argyrophilic grains: characteristic pathology of cerebral cortex in cases of adult onset dementia without Alzheimer changes. Neurosci Lett 76:124–127. 2438598 10.1016/0304-3940(87)90204-7

[8] Braak H , Braak E (1991) Neuropathological stageing of Alzheimer‐related changes. Acta Neuropathol 82:239–259. 1759558 10.1007/BF00308809

[9] Braak H , Braak E (1998) Argyrophilic grain disease: frequency of occurrence in different age categories and neuropathological diagnostic criteria. J Neural Transm 105:801–819. 9869320 10.1007/s007020050096

[10] Brettschneider J (2013) Stages of pTDP‐43 pathology in amyotrophic lateral sclerosis. Ann Neurol 74:20–38. 23686809 10.1002/ana.23937 PMC3785076

[11] Brettschneider J (2014) Sequential distribution of pTDP‐43 pathology in behavioral variant frontotemporal dementia (bvFTD). Acta Neuropathol 127:423–439. 24407427 10.1007/s00401-013-1238-y PMC3971993

[12] Cairns NJ , Bigio EH , Mackenzie IR , Neumann M , Lee VM , Hatanpaa KJ *et al* (2007) Neuropathologic diagnostic and nosologic criteria for frontotemporal lobar degeneration: consensus of the Consortium for Frontotemporal Lobar Degeneration. Acta Neuropathol 114:5–22. 17579875 10.1007/s00401-007-0237-2 PMC2827877

[13] Crary JF , Trojanowski JQ , Schneider JA , Abisambra JF , Abner EL , Alafuzoff I *et al* (2014) Primary age‐related tauopathy (PART): a common pathology associated with human aging. Acta Neuropathol 128:755–766. 25348064 10.1007/s00401-014-1349-0 PMC4257842

[14] Dan A , Takahashi M , Masuda‐Suzukake M , Kametani F , Nonaka T , Kondo H *et al* (2013) Extensive deamidation at asparagine residue 279 accounts for weak immunoreactivity of tau with RD4 antibody in Alzheimer's disease brain. Acta Neuropathol Commun 1:54. 24252707 10.1186/2051-5960-1-54 PMC3893535

[15] Dickson DW , Bergeron C , Chin SS , Duyckaerts C , Horoupian D , Ikeda K *et al* (2002) Office of rare diseases neuropathologic criteria for corticobasal degeneration. J Neuropathol Exp Neurol 61:935–946. 12430710 10.1093/jnen/61.11.935

[16] Feany MB , Dickson DW (1995) Widespread cytoskeletal pathology characterizes corticobasal degeneration. Am J Pathol 146:1388–1396. 7778678 PMC1870913

[17] Ferrer I , López‐González I , Carmona M , Arregui L , Dalfó E , Torrejón‐Escribano B *et al* (2014) Glial and neuronal tau pathology in tauopathies: characterization of disease‐specific phenotypes and tau pathology progression. J Neuropathol Exp Neurol 73:81–97. 24335532 10.1097/NEN.0000000000000030

[18] Forrest SL , Kril JJ , Wagner S , Hönigschnabl S , Reiner A , Fischer P *et al* (2019) Chronic traumatic encephalopathy (CTE) is absent from a European community‐based aging cohort while cortical aging‐related tau astrogliopathy (ARTAG) is highly prevalent. J Neuropathol Exp Neurol 78:398–405. 30939193 10.1093/jnen/nlz017

[19] Ghebremedhin E , Schultz C , Botez G , Rüb U , Sassin I , Braak E , Braak H (1998) Argyrophilic grain disease is associated with apolipoprotein E epsilon 2 allele. Acta Neuropathol 96:222–224. 9754952 10.1007/s004010050886

[20] Greenberg SG , Davies P (1990) A preparation of Alzheimer paired helical filaments that displays distinct tau proteins by polyacrylamide gel electrophoresis. Proc Natl Acad Sci U S A 87:5827–5831. 2116006 10.1073/pnas.87.15.5827 PMC54421

[21] Griggs RC , Wood DS (1989) Criteria for establishing the validity of genetic recombination in myotonic dystrophy. Neurology 39:420–421. 2927653 10.1212/wnl.39.3.420

[22] Haraguchi T , Ishizu H , Terada S , Takehisa Y , Tanabe Y , Nishinaka T *et al* (2000) An autopsy case of postencephalitic parkinsonism of von Economo type: some new observations concerning neurofibrillary tangles and astrocytic tangles. Neuropathology 20:143–148. 10935451 10.1046/j.1440-1789.2000.00287.x

[23] Hasegawa M , Watanabe S , Kondo H , Akiyama H , Mann DM , Saito Y *et al* (2014) 3R and 4R tau isoforms in paired helical filaments in Alzheimer’s disease. Acta Neuropathol 127:303–305. 24212601 10.1007/s00401-013-1191-9 PMC3895182

[24] Hattori M , Hashizume Y , Yoshida M , Iwasaki Y , Hishikawa N , Ueda R , Ojika K (2003) Distribution of astrocytic plaques in the corticobasal degeneration brain and comparison with tuft‐shaped astrocytes in the progressive supranuclear palsy brain. Acta Neuropathol 106:143–149. 12732936 10.1007/s00401-003-0711-4

[25] Hauw JJ , Daniel SE , Dickson D , Horoupian DS , Jellinger K , Lantos PL *et al* (1994) Preliminary NINDS neuropathologic criteria for Steele‐Richardson‐Olszewski syndrome (progressive supranuclear palsy). Neurology 44:2015–2019. 7969952 10.1212/wnl.44.11.2015

[26] Ikeda K , Akiyama H , Kondo H , Haga C , Tanno E , Tokuda T *et al* (1995) Thorn‐shaped astrocytes: possibly secondarily induced tau‐positive glial fibrillary tangles. Acta Neuropathol 90:620–625. 8615083 10.1007/BF00318575

[27] Ikeda C , Yokota O , Nagao S , Ishizu H , Oshima E , Hasegawa M *et al* (2016) The relationship between development of neuronal and astrocytic tau pathologies in subcortical nuclei and progression of argyrophilic grain disease. Brain Pathol 26:488–505. 26439704 10.1111/bpa.12319 PMC8029468

[28] Irwin DJ , Brettschneider J , McMillan CT , Cooper F , Olm C , Arnold SE *et al* (2016) Deep clinical and neuropathological phenotyping of Pick disease. Ann Neurol 79:272–287. 26583316 10.1002/ana.24559 PMC4755803

[29] Iwasaki Y , Yoshida M , Hattori M , Goto A , Aiba I , Hashizume Y , Sobue G (2004) Distribution of tuft‐shaped astrocytes in the cerebral cortex in progressive supranuclear palsy. Acta Neuropathol 108:399–405. 15365723 10.1007/s00401-004-0904-5

[30] Jellinger KA . (2001) Postencephalitic parkinsonism. In: Neurodegeneration: The Molecular Pathology of Dementia and Movement Disorders, 2nd edn, DW Dickson , RO Weller (eds.), Chapter 19, pp. 179–187. Wiley‐Blackwell Press: Oxford.

[31] Jellinger KA , Bancher C (1998) Senile dementia with tangles (tangle predominant form of senile dementia). Brain Pathol 8:367–376. 9546293 10.1111/j.1750-3639.1998.tb00160.x PMC8098213

[32] Jicha GA , Bowser R , Kazam IG , Davies P (1997) Alz‐50 and MC‐1, a new monoclonal antibody raised to paired helical filaments, recognize conformational epitopes on recombinant tau. J Neurosci Res 48:128–132. 9130141 10.1002/(sici)1097-4547(19970415)48:2<128::aid-jnr5>3.0.co;2-e

[33] Josephs KA , Murray ME , Whitwell JL , Tosakulwong N , Weigand SD , Petrucelli L *et al* (2016) Updated TDP‐43 in Alzheimer's disease staging scheme. Acta Neuropathol 131:571–585. 26810071 10.1007/s00401-016-1537-1 PMC5946692

[34] Koga S , Sanchez‐Contreras M , Josephs KA , Uitti RJ , Graff‐Radford N , van Gerpen JA *et al* (2017) Distribution and characteristics of transactive response DNA binding protein 43 kDa pathology in progressive supranuclear palsy. Mov Disord 32:246–255. 28009087 10.1002/mds.26809 PMC5933946

[35] Komori T , Arai N , Oda M , Nakayama H , Mori H , Yagishita S *et al* (1998) Astrocytic plaques and tufts of abnormal fibers do not coexist in corticobasal degeneration and progressive supranuclear palsy. Acta Neuropathol 96:401–408. 9797005 10.1007/s004010050911

[36] Kosaka K (1994) Diffuse neurofibrillary tangles with calcification: a new presenile dementia. J Neurol Neurosurg Psychiatry 57:594–596. 8201331 10.1136/jnnp.57.5.594 PMC1072922

[37] Kovacs GG (2018) Understanding the relevance of aging‐related tau astrogliopathy (ARTAG). Neuroglia 1:339–350.

[38] Kovacs GG , Ferrer I , Grinberg LT , Alafuzoff I , Attems J , Budka H *et al* (2016) Aging‐related tau astrogliopathy (ARTAG): harmonized evaluation strategy. Acta Neuropathol 131:87–102. 26659578 10.1007/s00401-015-1509-x PMC4879001

[39] Kovacs GG , Lee VM , Trojanowski JQ (2017) Protein astrogliopathies in human neurodegenerative diseases and aging. Brain Pathol 27:675–690. 28805003 10.1111/bpa.12536 PMC5578412

[40] Kovacs GG , Majtenyi K , Spina S , Murrell JR , Gelpi E , Hoftberger R *et al* (2008) White matter tauopathy with globular glial inclusions: a distinct sporadic frontotemporal lobar degeneration. J Neuropathol Exp Neurol 67:963–975. 18800011 10.1097/NEN.0b013e318187a80f PMC2785030

[41] Kovacs GG , Milenkovic I , Wöhrer A , Höftberger R , Gelpi E , Haberler C *et al* (2013) Non‐Alzheimer neurodegenerative pathologies and their combinations are more frequent than commonly believed in the elderly brain: a community‐based autopsy series. Acta Neuropathol 126:365–384. 23900711 10.1007/s00401-013-1157-y

[42] Kovacs GG , Molnár K , László L , Ströbel T , Botond G , Hönigschnabl S *et al* (2011) A peculiar constellation of tau pathology defines a subset of dementia in the elderly. Acta Neuropathol 122:205–222. 21437732 10.1007/s00401-011-0819-x

[43] Kovacs GG , Robinson JL , Xie SX , Lee EB , Grossman M , Wolk DA *et al* (2017) Evaluating the patterns of aging‐related tau astrogliopathy unravels novel insights into brain aging and neurodegenerative diseases. J Neuropathol Exp Neurol 76:270–288. 28340083 10.1093/jnen/nlx007 PMC6251691

[44] Kovacs GG , Xie SX , Robinson JL , Lee EB , Smith DH , Schuck T *et al* (2018) Sequential stages and distribution patterns of aging‐related tau astrogliopathy (ARTAG) in the human brain. Acta Neuropathol Commun 6:50. 29891013 10.1186/s40478-018-0552-y PMC5996526

[45] Kovacs GG , Yousef A , Kaindl S , Lee VM , Trojanowski JQ (2018) Connexin‐43 and aquaporin‐4 are markers of ageing‐related tau astrogliopathy (ARTAG)‐related astroglial response. Neuropahol Appl Neurobiol 44:491–505. 10.1111/nan.12427 PMC5788733 28755467

[46] Lasagna‐Reeves CA , Castillo‐Carranza DL , Sengupta U , Sarmiento J , Troncoso J , Jackson GR *et al* (2012) Identification of oligomers at early stages of tau aggregation in Alzheimer's disease. FASEB J 26:1946–1959. 22253473 10.1096/fj.11-199851 PMC4046102

[47] Mackenzie IR , Neumann M , Baborie A , Sampathu DM , Du Plessis D , Jaros E *et al* (2011) A harmonized classification system for FTLD‐TDP pathology. Acta Neuropathol 122:111–113. 21644037 10.1007/s00401-011-0845-8 PMC3285143

[48] Masuda‐Suzukake M , Nonaka T , Hosokawa M , Oikawa T , Arai T , Akiyama H *et al* (2013) Prion‐like spreading of pathological α‐synuclein in brain. Brain 136:1128–1138. 23466394 10.1093/brain/awt037 PMC3613715

[49] McKeith IG , Dickson DW , Lowe J , Emre M , O'Brien JT , Feldman H *et al* (2005) Diagnosis and management of dementia with Lewy bodies: third report of the DLB Consortium. Neurology 65:1863–1872. 16237129 10.1212/01.wnl.0000187889.17253.b1

[50] Miki T , Yokota O , Takenoshita S , Mori Y , Yamazaki K , Ozaki Y *et al* (2018) Frontotemporal lobar degeneration due to P301L tau mutation showing apathy and severe frontal atrophy but lacking other behavioral changes: A case report and literature review. Neuropahology 38:268–280. 10.1111/neup.12441 29105852

[51] Mirra SS , Heyman A , McKeel D , Sumi SM , Crain BJ , Brownlee LM *et al* (1991) The consortium to establish a registry for Alzheimer's disease (CERAD). Part II. Standardization of the neuropathologic assessment of Alzheimer's disease. Neurology 41:479–486. 2011243 10.1212/wnl.41.4.479

[52] Montine TJ , Phelps CH , Beach TG , Bigio EH , Cairns NJ , Dickson DW *et al* (2012) National Institute on Aging‐Alzheimer's Association guidelines for the neuropathologic assessment of Alzheimer's disease: a practical approach. Acta Neuropathol 123:1–11. 22101365 10.1007/s00401-011-0910-3 PMC3268003

[53] Munoz DG , Morris HR , Rossor M . (2001) Pick’s disease. In: Neurodegeneration: The Molecular Pathology of Dementia and Movement Disorders, 2nd edn, DW Dickson , RO Weller (eds.), Chapter 16, pp. 156–164. Wiley‐Blackwell Press: Oxford.

[54] Munoz DG , Neumann M , Kusaka H , Yokota O , Ishihara K , Terada S *et al* (2009) FUS pathology in basophilic inclusion body disease. Acta Neuropathol 118:617–627. 19830439 10.1007/s00401-009-0598-9

[55] Neumann M , Rademakers R , Roeber S , Baker M , Kretzschmar HA , Mackenzie IR (2009) A new subtype of frontotemporal lobar degeneration with FUS pathology. Brain 132:2922–2931. 19674978 10.1093/brain/awp214 PMC2768659

[56] Neumann M , Sampathu DM , Kwong LK , Truax AC , Micsenyi MC , Chou TT *et al* (2006) Ubiquitinated TDP‐43 in frontotemporal lobar degeneration and amyotrophic lateral sclerosis. Science 314:130–133. 17023659 10.1126/science.1134108

[57] Nishimura T , Ikeda K , Akiyama H , Kondo H , Kato M , Li F *et al* (1995) Immunohistochemical investigation of tau‐positive structures in the cerebral cortex of patients with progressive supranuclear palsy. Neurosci Lett 201:123–126. 8848233 10.1016/0304-3940(95)12151-x

[58] Nishimura M , Namba Y , Ikeda K , Oda M (1992) Glial fibrillary tangles with straight tubules in the brains of patients with progressive supranuclear palsy. Neurosci Lett 143:35–38. 1436679 10.1016/0304-3940(92)90227-x

[59] Nolan A , De Paula Franca Resende E , Petersen C , Neylan K , Spina S *et al* (2019) Astrocytic tau deposition is frequent in typical and atypical Alzheimer disease presentations. J Neuropathol Exp Neurol 78:1112–1123. 31626288 10.1093/jnen/nlz094 PMC7967845

[60] Okamoto K , Amari M , Fukuda T , Suzuki K , Takatama M (2019) Astrocytic tau pathologies in aged human brain. Neuropathology 39:187–193. 30937988 10.1111/neup.12544

[61] Papp MI , Kahn JE , Lantos PL (1989) Glial cytoplasmic inclusions in the CNS of patients with multiple system atrophy (striatonigral degeneration, olivopontocerebellar atrophy and Shy‐Drager syndrome). J Neurol Sci 94:79–100. 2559165 10.1016/0022-510x(89)90219-0

[62] Piao YS , Tan CF , Iwanaga K , Kakita A , Takano H , Nishizawa M *et al* (2005) Sporadic four‐repeat tauopathy with frontotemporal degeneration, parkinsonism and motor neuron disease. Acta Neuropathol 110:600–609. 16328530 10.1007/s00401-005-1086-5

[63] Saito Y , Ruberu NN , Sawabe M , Arai T , Tanaka N , Kakuta Y *et al* (2004) Staging of argyrophilic grains: an age‐associated tauopathy. J Neuropathol Exp Neurol 63:911–918. 15453090 10.1093/jnen/63.9.911

[64] Santpere G , Ferrer I (2009) Delineation of early changes in cases with progressive supranuclear palsy‐like pathology. Astrocytes in striatum are primary targets of tau phosphorylation and GFAP oxidation. Brain Pathol 19:177–187. 18462470 10.1111/j.1750-3639.2008.00173.x PMC8094872

[65] Schöll M , Lockhart SN , Schonhaut DR , O'Neil JP , Janabi M , Ossenkoppele R *et al* (2016) PET imaging of tau deposition in the aging human brain. Neuron 89:971–982. 26938442 10.1016/j.neuron.2016.01.028 PMC4779187

[66] Shibayama H , Kobayashi H , Nagasawa M , Yamada K , Iwata H , Iwai K *et al* (1992) Non‐Alzheimer non‐Pick dementia with Fahr’s syndrome. Clinical Neuropathol 11:237–250. 1424319

[67] de Silva R , Lashley T , Gibb G , Hanger D , Hope A , Reid A *et al* (2003) Pathological inclusion bodies in tauopathies contain distinct complements of tau with three or four microtubule‐binding repeat domains as demonstrated by new specific monoclonal antibodies. Neuropathol Appl Neurobiol 29:288–302. 12787326 10.1046/j.1365-2990.2003.00463.x

[68] Taniguchi‐Watanabe S , Arai T , Kametani F , Nonaka T , Masuda‐Suzukake M , Tarutani A *et al* (2016) Biochemical classification of tauopathies by immunoblot, protein sequence and mass spectrometric analyses of sarkosyl‐insoluble and trypsin‐resistant tau. Acta Neuroptahol 131:267–280. 10.1007/s00401-015-1503-3 PMC4713716 26538150

[69] Thal DR , Rüb U , Orantes M , Braak H (2002) Phases of A beta‐deposition in the human brain and its relevance for the development of AD. Neurology 58:1791–1800. 12084879 10.1212/wnl.58.12.1791

[70] Thal DR , Schultz C , Botez G , Del Tredici K , Mrak RE , Griffin WS *et al* (2005) The impact of argyrophilic grain disease on the development of dementia and its relationship to concurrent Alzheimer's disease‐related pathology. Neuropathol Appl Neurobiol 31:270–279. 15885064 10.1111/j.1365-2990.2005.00635.x

[71] The International Myotonic Dystrophy Consortium (IDMC) (2000) New nomenclature and DNA testing guidelines for myotonic dystrophy type 1 (DM1). Neurology 54:1218–1221. 10746587 10.1212/wnl.54.6.1218

[72] Tolnay M , Calhoun M , Pham HC , Egensperger R , Probst A (1999) Low amyloid (Abeta) plaque load and relative predominance of diffuse plaques distinguish argyrophilic grain disease from Alzheimer's disease. Neuropathol Appl Neurobiol 25:295–305. 10476046 10.1046/j.1365-2990.1999.00175.x

[73] Tolnay M , Probst A , Monsch AU , Staehelin HB , Egensperger R (1998) Apolipoprotein E allele frequencies in argyrophilic grain disease. Acta Neuropathol 96:225–227. 9754953 10.1007/s004010050887

[74] Trojanowski JQ , Revesz T ; Neuropathology Working Group on MSA (2007) Proposed neuropathological criteria for the post mortem diagnosis of multiple system atrophy. Neuropathol Appl Neurobiol 33:615–620. 17990994 10.1111/j.1365-2990.2007.00907.x

[75] Uchikado H , Lin WL , DeLucia MW , Dickson DW (2006) Alzheimer disease with amygdala Lewy bodies: a distinct form of alpha‐synucleinopathy. J Neuropathol Exp Neurol 65:685–697. 16825955 10.1097/01.jnen.0000225908.90052.07 PMC5706655

[76] Wharton SB , Minett T , Drew D , Forster G , Matthews F , Brayne C *et al* (2016) Epidemiological pathology of Tau in the ageing brain: application of staging for neuropil threads (BrainNet Europe protocol) to the MRC cognitive function and ageing brain study. Acta Neuropathol Commun 4:11. 26857919 10.1186/s40478-016-0275-x PMC4746919

[77] Yamada T , McGeer PL , McGeer EG (1992) Appearance of paired nucleated, Tau‐positive glia in patients with progressive supranuclear palsy brain tissue. Neurosci Lett 135:99–102. 1371861 10.1016/0304-3940(92)90145-w

[78] Yokota O , Terada S , Ishizu H , Tsuchiya K , Kitamura Y , Ikeda K *et al* (2002) NACP/alpha‐synuclein immunoreactivity in diffuse neurofibrillary tangles with calcification (DNTC). Acta Neuropathol 104:333–341. 12200618 10.1007/s00401-002-0545-5

[79] Yokota O , Tsuchiya K , Arai T , Yagishita S , Matsubara O , Mochizuki A *et al* (2009) Clinicopathological characterization of Pick's disease versus frontotemporal lobar degeneration with ubiquitin/TDP‐43‐positive inclusions. Acta Neuropathol 117:429–444. 19194716 10.1007/s00401-009-0493-4

[80] Yoshida K , Hata Y , Kinoshita K , Takashima S , Tanaka K , Nishida N (2017) Incipient progressive supranuclear palsy is more common than expected and may comprise clinicopathological subtypes: a forensic autopsy series. Acta Neuropathol 133:809–823. 28064358 10.1007/s00401-016-1665-7

