# Supplementary material for: Factors associated with development and distribution of granular/fuzzy astrocytes in neurodegenerative diseases
Source: Brain Pathol. 2020 May 6;30(4):811–30. doi: 10.1111/bpa.12843 (PMC7383906; doi:10.1111/bpa.12843)
Supplement: Supplementary file 1 — File S1 Supporting File S1. Demographic data of 18 PART cases with and without GFAs. [file BPA-30-811-s003.docx]

**Supporting File S1**

**Demographic data of 18 PART cases with and without GFAs**

All PART PART cases PART cases

cases with GFAs without GFAs

N (%) 18 7 (38.9) 11 (61.1)

Sex (male/female) 14/4 7/0 7/4

Age at death (y, mean±SD) 66.7±8.1 67.4±5.3 66.3±9.7

Brain weight (g, mean±SD) 1208.7±266.5 1286.7±194.7 1159.0±301.7

Braak stage [median (25-75 percentile)] 1.5 (1, 2) 2 (2, 2.5) 1 (1, 1.5)

Thal phase [median (25-75 percentile)] 0 (0, 0) 0 (0, 0) 0 (0, 0.5)

AGD stage [median (min, max) 0 (0, 0) 0 (0, 0) 0 (0, 0)

Cases having Lewy body disease [N, (%)] 1 0 (0.0) 1 (100.0)

Cases having TDP-43 pathology [N, (%)] 6 1 (16.7) 5 (83.3)

Pathological diagnosis

PART without any tauopathies or non-tauopathies [N, (%)] 5 4 (80.0) 1 (20.0)

PART with some non-tauopathy [N, %] 13 3 (23.1) 10 (76.9)

FTLD-TDP with PART [N, (%)] 2 0 (0.0) 2 (100.0)

ALS with PART [N, (%)] 4 1 (25.0) 3 (75.0)

MSA with PART [N, (%)] 5 1 (20.0) 4 (80.0)

DRPLA with PART [N, (%)] 1 1 (100.0) 0 (0.0)

Huntington’s disease with PART [N, (%)] 1 0 (0.0) 1 (100.0)

GFA: granular/fuzzy astrocyte, AGD: argyrophilic grain disease. Cases having TDP-43 pathology: all cases having FTLD-TDP, ALS-TDP, and other TDP-43-positive lesions in the limbic system and neocortex are included. PART: primary age-related tauopathy, pure definite PART: PART lacking not only Aβ deposits but also any other neurodegenerative diseases, FTLD-TDP: frontotemporal lobar degeneration with TDP-43-positive inclusions, ALS: amyotrophic lateral sclerosis, MSA: multiple system atrophy, DRPLA: dentatorubral-pallidoluysian atrophy.
