# Supplementary material for: Factors associated with development and distribution of granular/fuzzy astrocytes in neurodegenerative diseases
Source: Brain Pathol. 2020 May 6;30(4):811–30. doi: 10.1111/bpa.12843 (PMC7383906; doi:10.1111/bpa.12843)
Supplement: Supplementary file 2 — File S2 Supporting File S2. Distribution patterns of GFAs in AGD, PSP, AD, and PART cases. [file BPA-30-811-s002.docx]

**Supporting File S2**

**Distribution patterns of GFAs in AGD, PSP, AD, and PART cases**

AGD cases with GFAs (N = 26)

Striatum Amygdala Frontal cortex Stage^a)^ Number of cases (N)

- + - 1 4

+ + - 2a 6

- + + 2b 3

+ + + 3a or over 11

+ n.a. + unclassifiable 2

PSP cases with GFAs and TAs (N = 10)

Striatum Amygdala Frontal cortex Stage^b)^ Number of cases (N)

+ + + 3 or over 9

+ + - unclassifiable 1

AD cases with GFAs (N = 9)

Striatum Amygdala Frontal cortex Stage^c)^ Number of cases (N)

AD + - - 1 5

+ - + 2b 2

+ + + 3a or over 1

+ - - unclassifiable 1

PART cases with GFAs (N = 7)

Striatum Amygdala Frontal cortex Stage^a)^ Number of cases (N)

PART - + - 1 4

+ + + 3a or over 2

- n.a. + unclassifiable 1

GFA: granular/fuzzy astrocyte, TA: tufted astrocyte, AGD: argyrophilic grain disease, PSP: progressive supranuclear palsy, AD: Alzheimer’s disease, PART: primary age-related tauopathy, n.a.: not available. a) The staging system of pattern 2 for GFAs in the gray matter (amygdala first) proposed by Kovacs et al. [37, 44] was used for AGD and PART cases. b) For PSP cases, the staging system of sequential progression of combined GFAs and TAs in PSP [37, 44] was used. c) The staging system of pattern 1 was used for AD cases.
