# Supplementary material for: Factors associated with development and distribution of granular/fuzzy astrocytes in neurodegenerative diseases
Source: Brain Pathol. 2020 May 6;30(4):811–30. doi: 10.1111/bpa.12843 (PMC7383906; doi:10.1111/bpa.12843)
Supplement: Supplementary file 3 — File S3 Supporting File S3. Correlation of all cases. [file BPA-30-811-s001.docx]

**Supporting File S3**

**Correlation of all cases**

GFA GFA GFA GFA

(Frontal cortex) (Caudate nucleus) (Putamen) (Amygdala)

Age at death ρ 0.1804 0.2672 0.3584 0.1748

*P* 0.0655 0.0059* <0.001* 0.0884

Braak stage ρ 0.0026 0.0869 0.1082 -0.0971

*P* 0.9788 0.3783 0.2718 0.3466

Thal phase ρ 0.0286 0.1513 0.0959 -0.0768

*P* 0.7720 0.1235 0.3306 0.4569

AGD stage ρ 0.2239 0.2234 0.2705 0.5304

*P* 0.0217 0.0220 0.0053* <0.001*

Gallyas-positive TA (frontal cortex) ρ 0.6474 0.6091 0.5930 0.3727

*P* <0.001* <0.001* <0.001* <0.001*

Gallyas-positive TA (caudate nucleus) ρ 0.5343 0.5067 0.4953 0.2783

*P* <0.001* <0.001* <0.001* 0.0060*

Gallyas-positive TA (putamen) ρ 0.5464 0.5526 0.5341 0.3114

*P* <0.001* <0.001* <0.001* 0.0020*

Gallyas-positive TA (amygdala) ρ 0.3571 0.3368 0.3193 0.3317

*P* <0.001* <0.001* <0.001* <0.001*

AT8-positive TA (frontal cortex) ρ 0.6719 0.6104 0.5725 0.4142

*P* <0.001* <0.001* <0.001* <0.001*

AT8-positive TA (caudate nucleus) ρ 0.5939 0.6585 0.6399 0.3437

*P* <0.001* <0.001* <0.001* <0.001*

AT8-positive TA (putamen) ρ 0.5433 0.6053 0.5859 0.3614

*P* <0.001* <0.001* <0.001* <0.001*

AT8-positive TA (amygdala) ρ 0.4434 0.4368 0.4112 0.5972

*P* <0.001* <0.001* 0.0014* <0.001*

GFA: granular/fuzzy astrocyte, TA: tufted astrocyte, AGD: argyrophilic grain disease, ρ: Spearman rank order correlation coefficients. *: *P* <0.01.
